# Zeb1 sustains hematopoietic stem cell functions by suppressing mitofusin-2-mediated mitochondrial fusion

**DOI:** 10.1038/s41419-022-05194-w

**Published:** 2022-08-25

**Authors:** Kai Zhang, Huifang Zhao, Yaru Sheng, Xinyu Chen, Penghui Xu, Jinming Wang, Zhongzhong Ji, Yuman He, Wei-Qiang Gao, Helen He Zhu

**Affiliations:** 1 State Key Laboratory of Oncogenes and Related Genes, Renji-Med-X Stem Cell Research Center, Shanghai Cancer Institute, Ren Ji Hospital, Shanghai Jiao Tong University School of Medicine, 200127 Shanghai, China; 2 School of Biomedical Engineering & Med-X Research Institute, Shanghai Jiao Tong University, 200030 Shanghai, China; 3 Department of Urology, Ren Ji Hospital, Shanghai Jiao Tong University School of Medicine, 200127 Shanghai, China

**Keywords:** Haematopoietic stem cells, Stem-cell research

## Abstract

Metabolic status is essential in maintaining normal functions of hematopoietic stem cells (HSCs). However, how the dynamic of the mitochondrion, as a central organelle in metabolism, is molecularly regulated to orchestrate metabolism and HSC stemness remains to be elucidated. Here, we focus on the role of Zeb1, a well-characterized epithelial-to-mesenchymal transition (EMT) inducer which has been demonstrated to confer stem-cell-like characteristics in multiple cancer types in stemness regulation of HSCs. Using a *Zeb1-tdTomato* reporter mouse model, we find that Zeb1^+^Lin^−^Sca-1^+^c-Kit^+^ cells (Zeb1^+^-LSKs) represent a subset of functional long-term HSCs. Zeb1^+^LSKs exhibit a reduced reactive oxygen species (ROS) level, low mitochondrial mass, low mitochondrial membrane potential (MMP), and particularly small, round fragmented mitochondria. Of note, ectopic expression of Zeb1 leads to a fragmented mitochondrial morphology with a low mitochondrial metabolic status in EML cells. In addition, *Zeb1*-knockout (*Zeb1*-KO) LSKs from fetal liver display an exhausted stem-cell activity. *Zeb1* deficiency results in elongated and tubulated mitochondria with increased mitochondrial mass, elevated MMP, and higher ROS production. Mechanistically, Zeb1 acts as a transcriptional suppressor on the key mitochondrial-fusion protein Mitofusin-2 (encoded by *Mfn2*). We highlight an important role of Zeb1 in the regulation of mitochondrial morphology in HSC and the metabolic control of HSC stemness by repressing Mfn2-mediated mitochondrial fusion.

## Introduction

EMT has been extensively studied in cancer and found of particular importance in metastasis, therapeutic resistance, and stem-cell-like properties [1]. The EMT program is tightly regulated by key inducers, including the E-box binding motif transcription factor Zeb1, which has been reported to confer stem-cell-like characteristics in cancers [2–4]. Cancer stem-like cells (CSCs) are reminiscent of somatic stem cells (SSCs) in many aspects, not only in their dual capabilities of self-renewal and differentiation but in their relative quiescent state with low metabolic activities [5]. Although we and others have reported the role of Zeb1 in stem-cell-function maintenance and multilineage differentiation in prostate epithelial cells [6] and hematopoietic system [7, 8], the function of Zeb1 in orchestrating stemness maintenance and metabolic regulation in SSCs remains an intriguing question.

HSCs are responsible for hematopoiesis throughout their lifetime [9]. HSCs largely rely on glycolysis, featuring a low metabolic rate [10]. Under hematological stress, HSCs undergo a metabolic shift from glycolysis to oxidative phosphorylation (OXPHOS) before differentiation [11]. Metabolic reprogramming of HSCs is closely related to cell state and fate, including cell cycle exit and entry, stemness maintenance, and exhaustion [12]. However, how the metabolic status of HSC is molecularly regulated awaits further investigations.

Mitochondria play a central role in metabolism and connect metabolism to stem-cell functions [13]. Balanced mitochondrial morphology regulated by mitochondrial fusion and fission is vital for mitochondrial functions and stemness maintenance [14]. Mitochondrial-fusion proteins include Mitofusin-1 (Mfn1), Mitofusin-2 (Mfn2), and Opa1 [15–17]. They fuse the outer and inner membranes between neighboring mitochondria, thereby conferring a tubulated mitochondrial network [14, 18]. Conversely, mitochondrial-fission-proteins, including Drp1 and Fis1, divide larger mitochondria into smaller and round-shaped ones, promoting a fragmented mitochondrial network [14, 19]. These mitochondrial dynamic molecules are crucial in stem-cell functions in distinct contexts [20, 21]. Despite that important progress, how the dynamics of the mitochondrion are molecularly regulated to orchestrate HSC metabolism and stemness remains elusive.

Here, we reveal that the EMT inducer Zeb1 links a fragmented mitochondrial morphology to HSC stemness by suppressing Mitofusin-2, thereby sustaining immature mitochondrial machinery and a low mitochondrial metabolic state. Our results highlight an important role of Zeb1/Mitofusin-2/fragmented mitochondria axis in orchestrating mitochondrial status and long-term self-renewal properties in HSCs.

## Results

### *Zeb1* is positively associated with stem-cell signature in the hematopoietic system

**Fig. 1:**
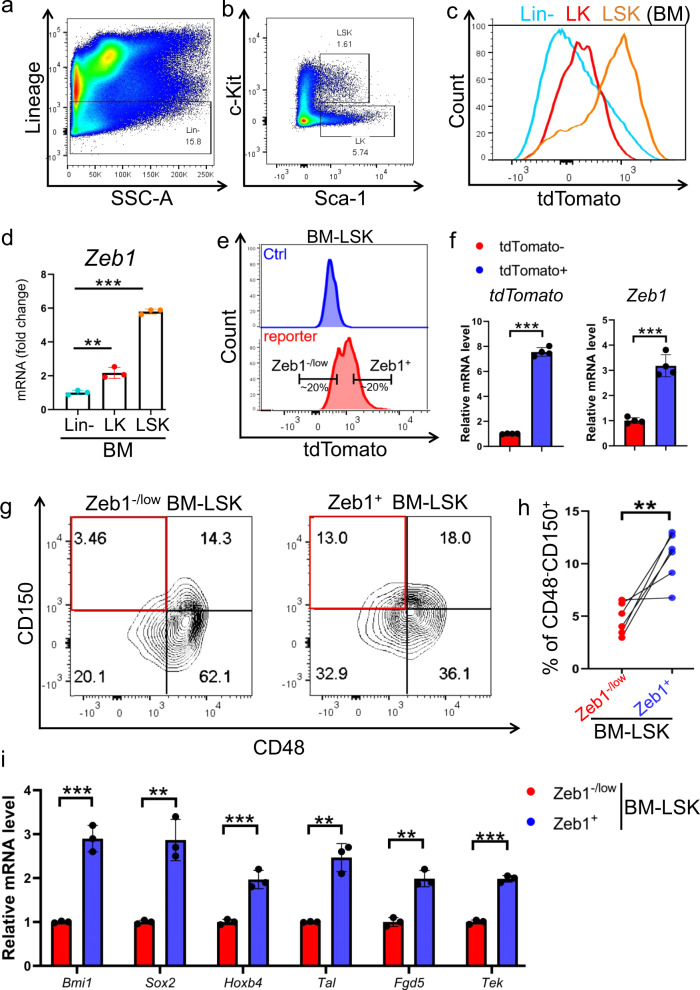
Zeb1 is enriched in the HSC-containing BM-LSK subset. **a, b** Lineage negative (Lin^−^) cells (**a**) were gated from the BM cells of *Zeb1-tdTomato* reporter mice (male, 8 week old), and Lin^−^c-Kit^+^ (LK) and Lin^−^Sca1^+^c-Kit^+^ (LSK) cells (**b**) were shown. 2 × 10^7^ total bone marrow (BM) cells were collected from each mouse, the immature Lin^−^ cells take up to 15–20% of total BM cells. Two well-characterized immunophenotypic markers Sca-1 (APC) and c-Kit (Alexa Fluor-700) further distinguish progenitor cells enriched Lin^−^c-Kit^+^ (LK) and HSC-enriched Lin^-^Sca-1^+^c-Kit^+^ (LSK) cells. The LK and LSK subsets take up to around 5–8% and 1.5–2.5%, respectively. **c** Flow cytometric analysis showing the fluorescent intensity of tdTomato in Lin^−^, LK, and LSK subsets. **d** RT-qPCR data validating the Zeb1 expression level in Lin^−^, LK, and LSK subsets. Results are shown as mean ± SD. **p < 0.001; ***P < 0.0001. These data were collected from 3 *Zeb1-tdTomato* reporter mice. **e** The right side 20% LSKs with the maximal tdTomato fluorescence intensity were identified as Zeb1^+^ BM-LSK cells (Zeb1^+^-LSKs). The left side 20% LSK with the minimal tdTomato fluorescence intensity were defined as Zeb1^−/low^ BM-LSK cells (Zeb1^−/low^-LSKs) using flow cytometry (lower panel, red color). The LSK cells from WT mice with no tdTomato fluorescence were used as a control (upper panel, blue color). **f** RT-qPCR results confirming that *Zeb1* is significantly elevated in tdTomato^+^ BM-LSK cells as compared to tdTomato^−^ counterparts. Data are represented as mean ± SD. ***P < 0.0001. These data were collected from 3 *Zeb1-tdTomato* reporter mice. **g, h** Frequencies of CD150^+^CD48^−^LSK long-term HSC in Zeb1^−/low^-BM-LSK (left panel) and Zeb1^+^-BM-LSKs (right panel) (**g**). Quantification results were from 6 *Zeb1-tdTomato* reporter mice (**h**). **i** RT-qPCR results showing the relative expression level of stem-cell-associated-genes including *Bmi1*, *Sox2*, *Hoxb4*, *Tal1*, *Fgd5*, and *Tek* in Zeb1^−/low^ and Zeb1^+^-LSKs. Data are represented as mean ± SD. **p < 0.001; ***P < 0.0001. These data were collected from 3 *Zeb1-tdTomato* reporter mice.

To explore the biological relevance of Zeb1 expression in HSC function at the physiological level, we employed the *Zeb1-tdTomato*-reporter mouse model reported in our previous study [6]. Briefly, a *tdTomato* cassette was inserted behind exon 8 of *Zeb1* gene via CRISPR/Cas9 technology (Supplementary Fig. 1a), by which the *tdTomato* expression was controlled by endogenous *Zeb1* transcription. These mice were validated by PCR-based genotyping (Supplementary Fig. 1b). Using a lineage cocktail flow antibody including B220, CD3e, Gr-1, Mac-1, and Ter119, we can distinct mature blood cells and immature lineage negative (Lin^−^) bone marrow (BM) cells (Fig. 1a). In Lin^−^ population, two well-established immunophenotypic markers Sca-1 and c-Kit further distinguished progenitor cells enriched Lin^−^c-Kit^+^ (LK) and HSC-enriched Lin^−^Sca-1^+^c-Kit^+^ (LSK) cells (Fig. 1b). The gating strategy is shown in Supplementary Fig. 1c–f. We assessed Zeb1 levels in Lin^−^, LK, and LSK BM cells. Intriguingly, LSKs exhibited the highest expression of Zeb1, as reflected by the strongest tdTomato fluorescence intensity (Fig. 1c). RT-qPCR results also confirmed that Zeb1 mRNA is highest in FACS-sorted LSKs (Fig. 1d), suggesting that Zeb1 level is positively associated with stem-cell feature in the hematopoietic system.

We further interrogated the biological relevance of Zeb1 level in HSC-enriched BM-LSK cells. We defined the right side 20% tdTomato^+^ LSK with the maximal fluorescence intensity as Zeb1^+^-LSKs, while the left side 20% cells with the minimal fluorescence value as Zeb1^-/low^-LSKs (Fig. 1e). RT-qPCR data validated that the Zeb1 levels in tdTomato^+^ BM-LSKs were significantly higher than tdTomato^-^ counterparts, suggesting tdTomato fluorescence faithfully reflects the Zeb1 level in BM-LSKs (Fig. 1f).

Since CD48^−^CD150^+^LSK are regarded as long-term HSCs (LT-HSCs) [9], we assessed the enrichment of HSC in Zeb1^+^ and Zeb1^−/low^-LSKs. Approximately 13.0% of Zeb1^+^-LSKs were positive for HSCs, in contrast, there was merely 5% of LT-HSC in Zeb1^−/low^-LSKs (Fig. 1g). The quantitative results from 6 *Zeb1-tdTomato*-reporter mice are shown in Fig. 1h, suggesting a significantly higher enrichment of LT-HSC in the Zeb1^+^-LSKs than in the Zeb1^−/low^ counterparts. Next, we assessed the expression of stem-cell-associated molecules in Zeb1^+^ and Zeb1^−/low^-LSKs. *Bmi1*, *Sox2*, *Hoxb4*, *Tal1*, *Fgd5*, and *Tek1* were significantly elevated in the Zeb1^+^-LSKs compared with the Zeb1^−/low^ cells (Fig. 1i), suggesting that Zeb1 expression is positively correlated with the HSC signature in BM-LSKs.

### Zeb1^+^-LSKs exhibit enhanced self-renewal and multilineage reconstitution capabilities

**Fig. 2:**
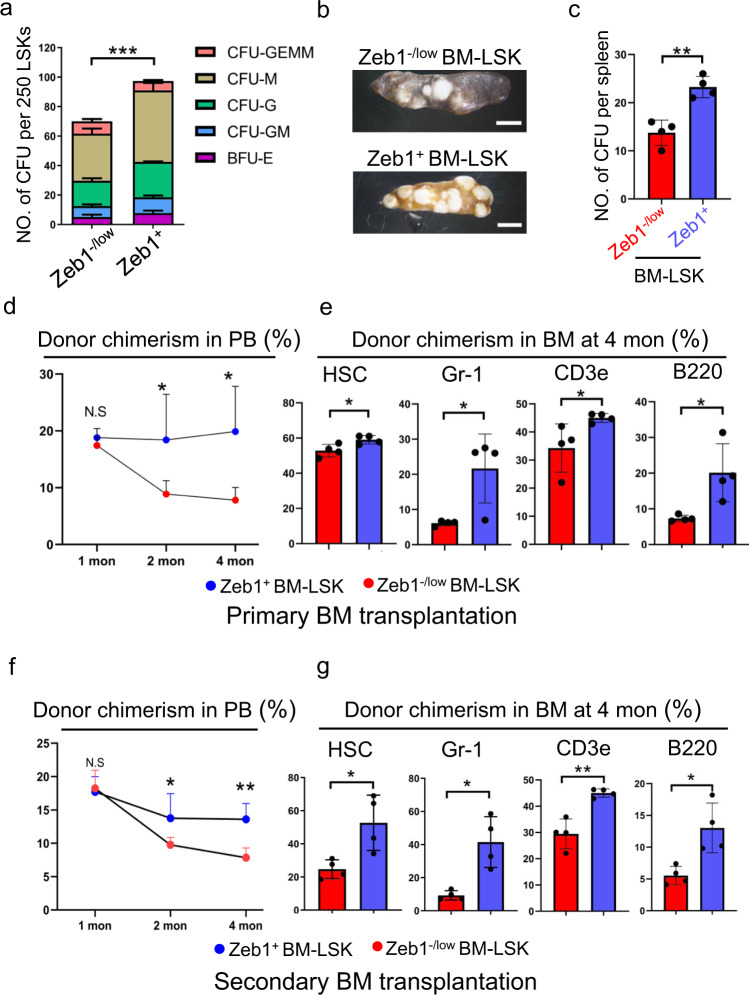
Zeb1^+^-LSKs exhibit stronger stem cells-activity than their Zeb1^-/low^ counterparts. **a** The in vitro CFU assay showing the colony numbers of BFU-E, CFU-GM, CFU-G, CFU-M, and CFU-GEMM derived from 250 Zeb1^−/low^ and Zeb1^+^-LSKs. BFU-E: burst-forming unit-erythrocyte; CFU-E CFU- erythrocyte, CFU-G CFU-granulocyte, CFU-M CFU-macrophage, CFU-GEMM CFU-granulocyte, erythrocyte, macrophage, megakaryocyte. ***P < 0.0001. **b, c** The in vivo CFU-spleen (CFU-S) assay showing splenic nodules derived from Zeb1^−/low^ and Zeb1^+^-LSKs (**b**). Quantification results from 4 *Zeb1-tdTomato* reporter mice were presented (**c**). **p < 0.001. **d** The donor chimerism curves showing the blood reconstitution contributed by Zeb1^−/low^ and Zeb1^+^-LSK donor cells in PB at indicated time points in the primary BM transplantation. *p < 0.01, N.S no significance. **e** Chimerism contributed by Zeb1^−/low^ and Zeb1^+^-LSK donor cells from BM showing the multilineage reconstitution potential in the PB, including HSC (CD150^+^CD48^−^LSK); myeloid cells (Gr-1^+^); T cell (CD3e^+^) and B cell (B220^+^) were determined. Data are represented as mean ± SD. *p < 0.01; **p < 0.001; ***P < 0.0001. **f** The donor chimerism curves showing the blood reconstitution contributed by Zeb1^−/low^ and Zeb1^+^-LSK donor cells in the PB at indicated time points in the secondary BM transplantation. Data are represented as mean ± SD. *p < 0.01; **p < 0.001, N.S no significance. **g** Chimerism contributed by Zeb1^−low^ and Zeb1^+^-LSK donor cells in BM showing the multilineage reconstitution potential of Zeb1^−/low^ and Zeb1^+^-LSKs including HSC (CD150^+^CD48^-^LSK), myeloid cells (Gr-1^+^), T cell (CD3e^+^), and B cell (B220^+^). Data are represented as mean ± SD. *p < 0.01; **p < 0.001.

Next, we performed in vitro colony-forming-unit (CFU) assays [22] to functionally determine the hematopoietic activity of the Zeb1^+^ and Zeb1^−/low^-LSKs in producing multipotent progenitors, such as CFU-granulocyte-erythrocyte-monocyte-megakaryocyte (CFU-GEMM), burst-forming-unit-erythroid (BFU-E), CFU-granulocyte/monocyte (CFU-GM), CFU-granulocyte (CFU-G), and monocyte/macrophage (CFU-M). The Zeb1^+^-LSKs displayed significantly higher efficiency in generating these colonies than Zeb1^−/low^ counterparts (Fig. 2a). We also performed in vivo CFU-spleen (CFU-S) experiments. Zeb1^+^ and Zeb1^−/low^-LSKs were intravenously injected into host mice. LSK-derived nodular colonies in the spleen at 14 days were enumerated (Fig. 2b). The Zeb1^+^-LSKs gave rise to many more colonies than the Zeb1^−/low^-LSKs. The colony number was quantified in Fig. 2c.

To determine the long-term self-renewal and multilineage activity of the Zeb1^+^ and Zeb^−/low^-LSKs, two rounds of competitive BM reconstitution experiments were performed (Supplementary Fig. 2a). 200 Zeb1^+^ and Zeb^−/low^-LSKs were FACS-sorted from *Zeb1*-*tdTomato*-reporter mice (CD45.2) and mixed with 2 × 10^5^ BM competitor cells (CD45.1). These mixed donor/competitor cells were then transplanted into X-ray lethally irradiated recipients (CD45.1). The gating strategies for FACS analysis of peripheral blood (PB) chimerism are shown in Supplementary Fig. 2b. We monitored the chimerism by Zeb1^+^ and Zeb^−/low^-LSK donor cells in PB at indicated time points (Fig. 2d). The contribution of the Zeb1^+^-LSKs to PB chimerism was significantly higher than that of their Zeb1^−/low^ counterparts. We also assessed BM reconstitution by Zeb1^+^ and Zeb^−/low^-LSKs in the 4th month. Compared to Zeb1^+^-LSKs, the Zeb^−/low^ cells displayed significantly reduced lineage reconstitution in BM, including HSCs, myeloid cells, T, and B lymphoid cells (Fig. 2e).

We then performed secondary BM transplantation experiments. Zeb1^+^-LSKs showed sustainable advantages in multilineage reconstitution over Zeb1^−/low^ subsets in PB (Fig. 2f). The reconstitution of HSCs and mature lineage cells by Zeb1^+^-LSKs was also significantly higher than that of the Zeb1^−/low^ cells (Fig. 2g).

**Fig. 3:**
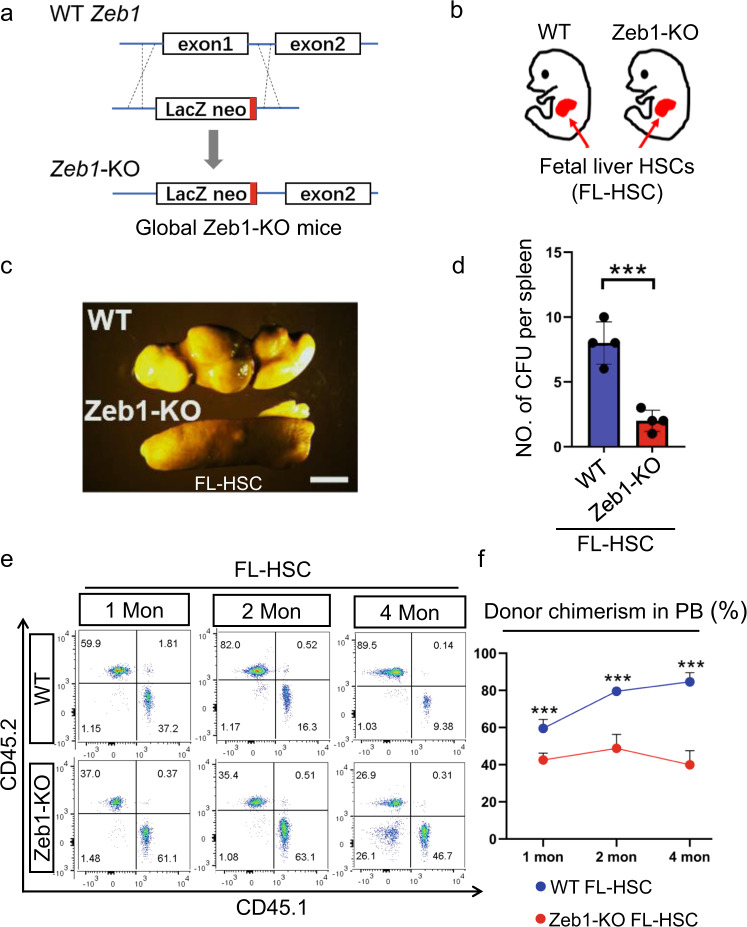
Acute loss of Zeb1 in the fetal liver HSCs leads to reduced self-renewal and multilineage reconstitution capabilities. **a** The schematic strategy of generation of global *Zeb1*-KO mice, in which, exon 1 was replaced with a *Lac-Z* neo fragment including a terminal signal. **b** The fetal liver HSCs (FL-HSCs) were purified from *Zeb1*-KO mice and their WT littermates at E13.5 due to embryonic lethality. **c** In vivo CFU-S experiments showing the splenic nodules derived from WT and *Zeb1*-KO FL-HSCs. **d** Quantification of splenic nodules derived from FL-HSCs from WT and *Zeb1*-KO mice (*n* = 4). Data are represented as mean ± SD. ***p < 0.0001. **e, f** The represented flow cytometric data (**e**) and the donor chimerism curves (**f**) showing the chimerism in the PB contributed by WT and *Zeb1*-KO FL-HSC donor cells at indicated time points after BM transplantation. *n* = 5, Data are represented as mean ± SD. ***p < 0.0001.

We further assessed whether acute loss-of-*Zeb1* would affect the stem-cell activity and multilineage differentiation in HSCs using global *Zeb1*-knockout (*Zeb1*-KO) mice (Fig. 3a). Due to the embryonic lethality of the *Zeb1*-KO mice, we assessed fetal liver HSCs (FL-HSCs) from *Zeb1*-KO and wild-type (WT) littermates at E13.5 (Fig. 3b). The *Zeb1*-KO FL-HSCs exhibited significantly fewer splenic colonies than WT counterparts (Fig. 3c, d). FL-HSCs from WT (CD45.2) and Zeb1-KO (CD45.2) mice were transplanted into X-ray lethally irradiated CD45.1 recipients (Supplementary Fig. 2c). Interestingly, acute loss-of-*Zeb1* resulted in significantly decreased blood reconstitution in PB (Fig. 3e–f). These results were in line with those from the *Zeb1-tdTomato*-reporter mice, suggesting a broad implication of the role of Zeb1 in long-term self-renewal and sustainable hematopoiesis of both adult and fetal HSCs.

### Zeb1^+^-LSKs exhibit low mitochondrial mass and low MMP and ROS production

**Fig. 4:**
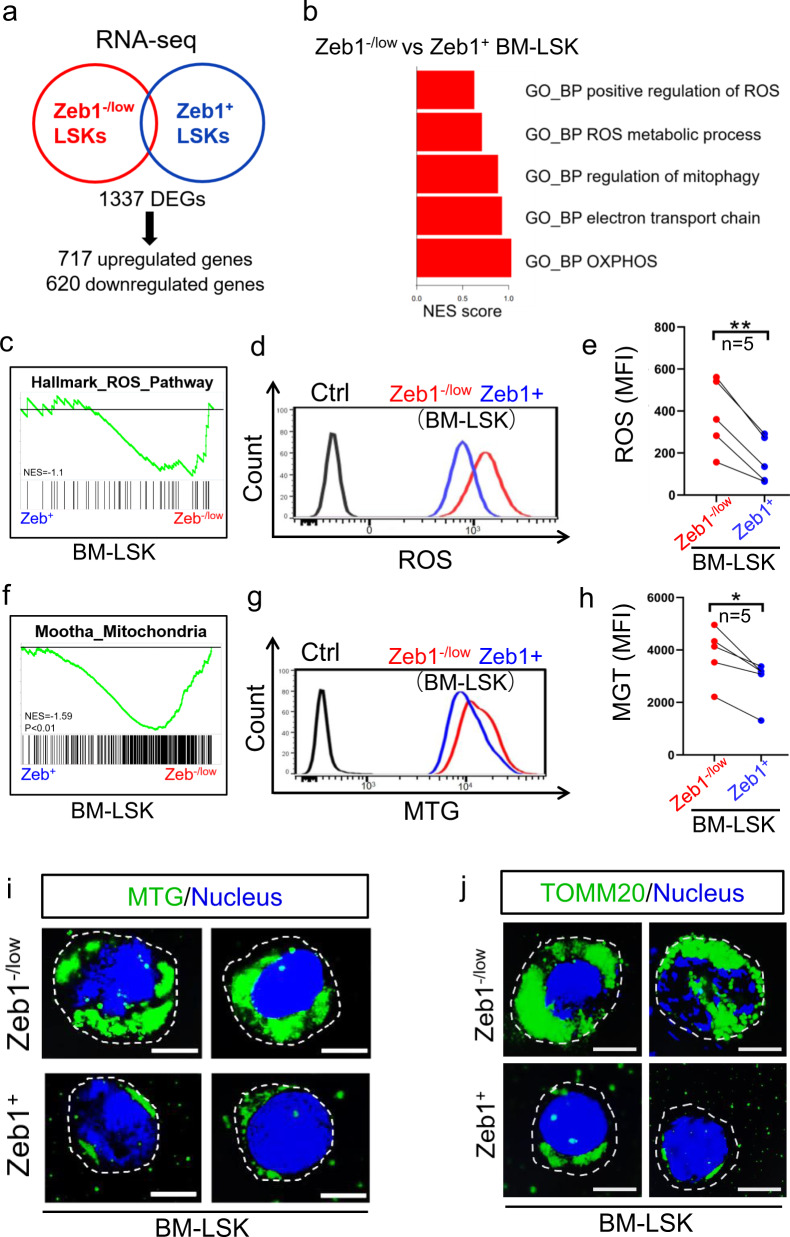
Zeb1^+^ LSKs exhibit low mitochondrial mass and lower MMP and ROS production. **a** RNA-seq performed on FACS-sorted Zeb1^−/low^ versus Zeb1^+^-LSKs reveals 1337 DEGs including 717 upregulated genes and 620 downregulated genes. **b** Gene Ontology (GO) analysis shows the top 5 biological process (GO_BP) in Zeb1^−/low^ versus Zeb1^+^-LSKs. **c** Gene set enrichment analysis (GSEA) plots showing the enrichment of Hallmark of ROS pathway in Zeb1^−/low^-LSKs as compared to Zeb1^+^-LSKs. **d, e** Representative FACS plot (**d**) and median fluorescence intensity (MFI) (**e**) of ROS of Zeb1^−/low^ and Zeb1^+^-LSKs using a H2DCFDA probe. Data were collected from 5 *Zeb1-tdTomato* reporter mice. **p < 0.001. **f** The GSEA plots showing the enrichment of Mitochondria signature in Zeb1^−/low^-LSKs as compared to Zeb1^+^-LSKs. **g, h** Representative FACS plot (**g**) and median fluorescence intensity (MFI) (**h**) of Mito-Tracker-Green (MTG) of Zeb1^−/low^ and Zeb1^+^-LSKs using 5 reporter mice. *p < 0.01. **i, j** Immunofluorescence (IF) images of MTG (**i**) and TOMM20 (**j**) in FACS-sorted Zeb1^−/low^ and Zeb1^+^-LSKs. Images were captured using a structured illumination microscope (SIM). The nucleus was counterstained with Hoechst dye. Scale bar = 5 μm.

To dissect the mechanism by which Zeb1^+^-LSKs exhibit stronger capabilities of self-renewal and multilineage reconstitution than the Zeb1^−/low^-LSKs. We performed RNA sequencing (RNA-seq) on FASC-sorted Zeb1^+^ and Zeb^−/low^-LSKs. The differentially expressed genes (DEGs) were identified in the Zeb1^−/low^ versus Zeb1^+^-LSKs (Fig. 4a). A Gene Ontology (GO) analysis of the upregulated genes demonstrated that ROS, electron transport chain (ETC), and OXPHOS-related biological processes were most significantly enriched in the Zeb1^−/low^ versus Zeb1^+^-LSKs (Fig. 4b). Consistent with the lower ROS and OXPHOS signatures in the Zeb1^+^-LSKs, a gene set enrichment analysis (GSEA) revealed decreased ROS hallmarks in the Zeb1^+^-LSKs (Fig. 4c). To validate these results, we assessed the intracellular ROS levels in Zeb1^−/low^ and Zeb1^+^-LSKs using a fluorescent probe. Consistently, the Zeb1^+^-LSKs showed lower ROS levels (Fig. 4d) reflected by significantly reduced median fluorescence intensity (MFI) (Fig. 4e) than Zeb1^−/low^ cells. Consistently, GSEA results showed a reduced mitochondrial signature in the Zeb1^+^-LSKs (Fig. 4f), suggesting that Zeb1^+^-LSKs may have less mitochondrial mass or a less functional mitochondrial machinery. To validate this, we determined the mitochondrial mass in the Zeb1^−/low^ and Zeb1^+^-LSKs using the Mito-tracker-Green (MTG) probe. Indeed, the Zeb1^+^-LSKs had lower mitochondrial mass (Fig. 4g) with weaker MTG MFI (Fig. 4h) than Zeb1^−/low^-LSKs. Immunofluorescence (IF) of MTG (Fig. 4i) and the mitochondrial outer membrane molecule TOMM20 (Fig. 4j) confirmed that Zeb1^+^-LSKs have lower mitochondrial mass than their Zeb1^−/low^ counterparts. These data demonstrated that the lower mitochondrial mass in the Zeb1^+^-LSKs might contribute to a lower mitochondrial metabolism with lower ROS production, and reduced ETC and OXPHOS activity, which benefit stronger self-renewal and multilineage reconstitution capacities in BM-HSCs.

HSCs restrict mitochondrial respiration and mainly rely on glycolysis for their maintenance. We sought to assess whether Zeb1^+^-LSKs are prone to utilize glycolysis for energy production. Indeed, most of the key glycolytic enzymes were upregulated in Zeb1^+^-LSKs versus Zeb1^−/low^ cells (Supplementary Fig. 3a). The RT-qPCR results validated glycolytic enzymes including *Pfkl*, *Aldoc*, *Gapdh*, and *Pgm2* were significantly higher in Zeb1^+^-LSKs than Zeb1^−/low^ counterparts (Supplementary Fig. 3b). The GSEA plot revealed increased glycolysis hallmarks in the Zeb1^+^-LSKs rather than Zeb1^−/low^ cells (Supplementary Fig. 3c). Although their glucose-uptake rates were comparable (Supplementary Fig. 3d), Zeb1^+^-LSKs displayed higher lactate secretion than Zeb1^−/low^ cells (Supplementary Fig. 3e), confirming that Zeb1^+^-LSKs mainly utilize glycolysis for energy metabolism. The elevated glycolysis in Zeb1^+^-LSKs may explain why they have a lower amount of ROS than Zeb1^−/low^-LSKs. As excessive ROS is detrimental to HSCs, we found that ROS scavengers, including superoxide dismutase (*Sod1/2*) and catalase (*Cat*), were significantly higher in Zeb1^+^-LSKs than those in Zeb1^−/low^ cells (Supplementary Fig. 3f, g), suggesting a stronger antioxidant activity that facilitates stem-cell function in Zeb1^+^-LSKs.

**Fig. 5:**
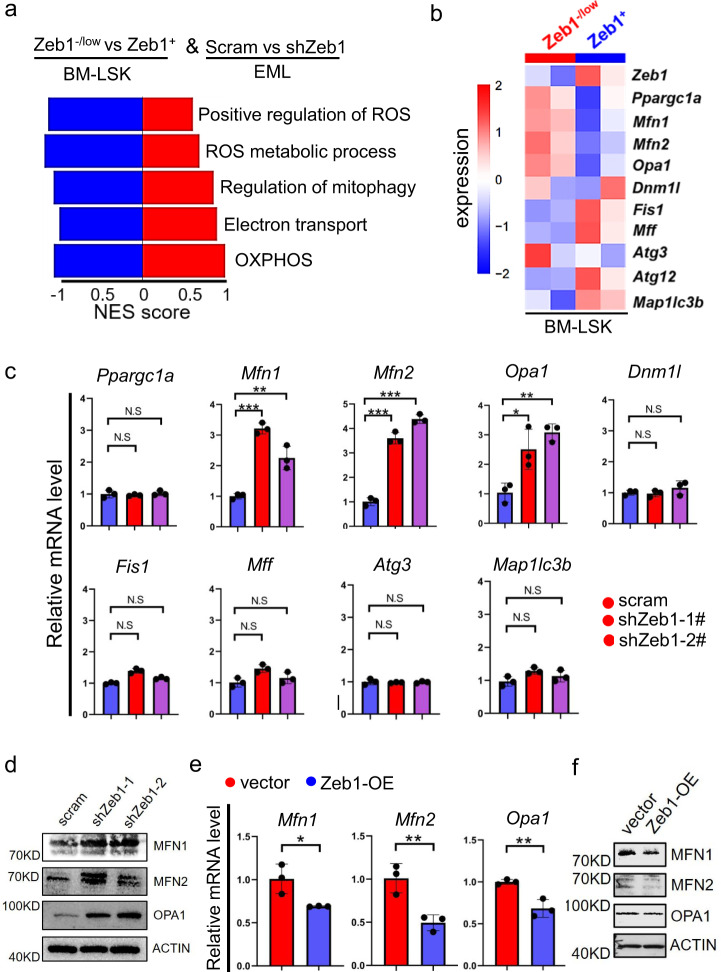
Zeb1 suppresses mitochondrial fusion in EML cells. **a** GO analysis showing the top 5 biological process based on the overlapped DEGs from BM-LSKs (Zeb1^−/low^ and Zeb1^+^-LSKs) and EMLs (EML-scramble and EML-shZeb1) cells. **b** Heatmap showing the expression of mitochondrial dynamic related genes in the Zeb1^−/low^ and Zeb1^+^-LSKs. **c** RT-qPCR results showing the alteration of mitochondrial dynamic related genes in response to the downregulation of Zeb1 in EML cells. Data are represented as mean ± SD. *p < 0.01; **p < 0.001; ***P < 0.0001. **d** Immunoblots showing the indicated proteins in EML-scramble, shZeb1-1# and shZeb1-2# cells. **e** RT-qPCR results showing the alteration of mitochondrial-fusion-related genes in response to ectopic expression of Zeb1 in EML cells. Data are represented as mean ± SD. *p < 0.01; **p < 0.001. **f** Immunoblots of the indicated proteins in EML-vector and Zeb1-OE cells.

To assess whether Zeb1 can directly confer cells with a lower mitochondrial metabolic status, we overexpressed Zeb1 (Zeb1-OE) in EML cells, a hematopoietic progenitor cell line (Supplementary Fig. 4a, b). We found that the MTG MFI was significantly decreased in EML-Zeb1-OE cells compared to vector cells (Supplementary Fig. 4c). A tetra-methyl-rhodamine-ethyl-ester (TMRE) dye was used to assess the mitochondrial membrane potential (MMP), and EML-Zeb1-OE cells showed a lower TMRE MFI than vector cells (Supplementary Fig. 4d). Next, we determined the ROS levels of EML-Zeb1-OE and vector cells. A significantly lower ROS level was detected in the EML-Zeb1-OE cells than in vector cells (Supplementary Fig. 4e). We then established two stable EML lines in which Zeb1 was significantly downregulated by two shRNAs. The knockdown efficiencies of EML-shZeb1 cells were validated by RT-qPCR and immunoblotting assays (Supplementary Fig. 5a-b). We performed RNA-seq on EML-scramble and shZeb1 cells to identify their DEGs. These two cohorts of DEGs in Zeb1^−/low^ versus Zeb1^+^-LSKs (Fig. 4a) and EML-scramble versus shZeb1 cells were combined for analysis. The overlapped DEGs of these two cohorts were also enriched in ROS, ETC, and OXPHOS-related signals (Fig. 5a). The mitochondrial mass (Supplementary Fig. 5c), MMP (Supplementary Fig. 5d), and ROS (Supplementary Fig. 5e) levels were all increased in EML-shZeb1 cells versus scramble cells. Next, we treated EML-shZeb1 cells with the n-acetyl-cysteine (NAC), a ROS scavenger, and found that stem-cell-associated-genes (Supplementary Fig. 5f–h) were significantly increased in response to NAC. These results suggested that Zeb1-mediated HSC stemness maintenance is closely associated with a less functional mitochondrial machinery, reflected by decreased MMP and lower ROS levels.

### Zeb1 confers a low mitochondrial metabolism by suppressing mitochondrial fusion

Mitochondrial function is associated with mitochondrial mass and morphology [23]. We next sought to delineate the mechanism by which overexpression of Zeb1 suppresses mitochondrial mass and MMP. Decreased mitochondrial mass and MMP might be due to decreased mitochondrial biogenesis, enhanced mitophagy, increased mitochondrial fission, or reduced mitochondrial fusion. We assessed the expression of the key mitochondrial-biogenesis-related-gene (*Ppargc1a*), mitochondrial-fusion molecules (*Mfn1*, *Mfn2,* and *Opa1*), mitochondrial-fission-genes (*Dnm1l*, *Fis1,* and *Mff*) and mitophagy-associated-genes (*Atg3*, *Atg12,* and *Map1lc3b*) in the Zeb1^−/low^ and Zeb1^+^-LSKs. The heatmap showed that *Ppargc1a*, *Mfn1*, *Mfn2,* and *Opa1* were all decreased in the Zeb1^+^ LSKs versus Zeb1^−/low^ counterparts (Fig. 5b). To validate these results in EML cells, we performed RT-qPCR experiments in EML-scramble and shZeb1 cells. The RT-qPCR (Fig. 5c) and immunoblotting results (Fig. 5d) showed that only *Mfn1*, *Mfn2,* and *Opa1* were significantly upregulated when *Zeb1* is downregulated. Conversely, *Mfn1*, *Mfn2,* and *Opa1* were significantly decreased in EML-Zeb1-OE cells at both mRNA (Fig. 5e) and protein (Fig. 5f) levels. These results suggested that *Zeb1* may repress mitochondrial fusion.

### Zeb1 endows EML cells with a fragmented mitochondrial morphology

**Fig. 6:**
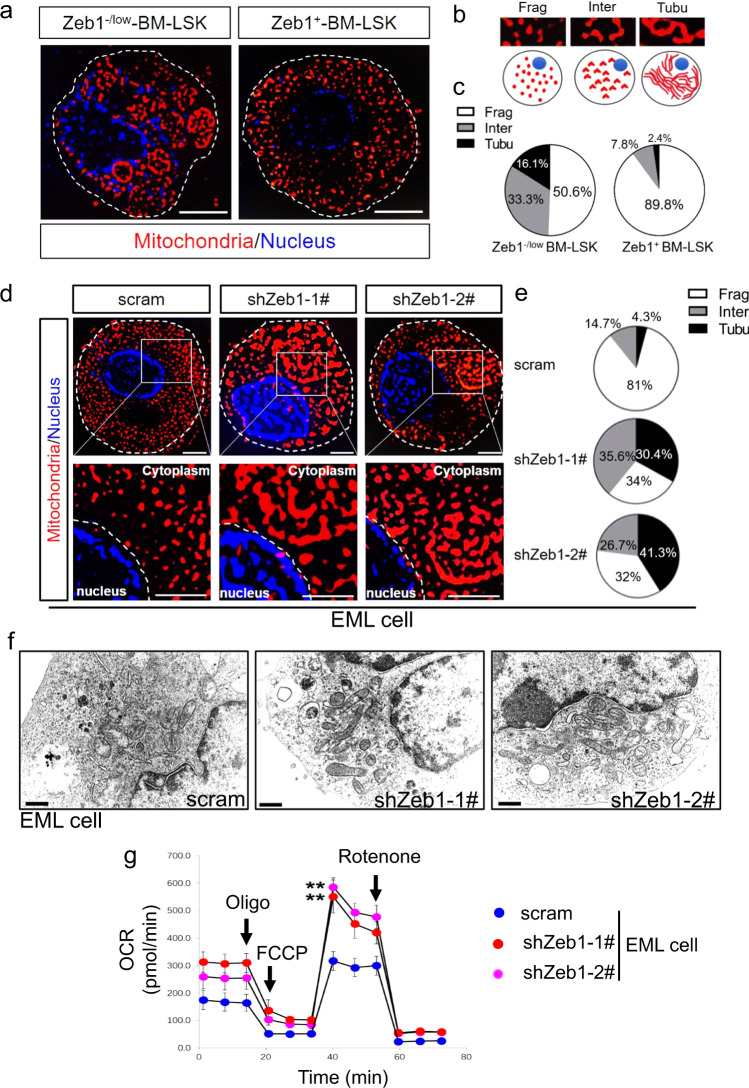
Zeb1 confers fragmented mitochondria to EML cells. **a** Representative IF images of Mito-Tracker-Red in BM Zeb1^−/low^ and Zeb1^+^-LSKs from *Zeb1-tdTomato* reporter mice. All images were captured using a SIM microscope. The nucleus was counterstained with Hoechst dye. Scale bar = 3 μm. **b** Typical structures of fragmented (Frag) and tubulated (Tubu) and intermediate (Inter) mitochondria. **c** Quantifications of Frag, Tubu and Inter mitochondria in BM Zeb1^−/low^ and Zeb1^+^-LSKs. **d** IF images of Mito-Tracker-Red in EML-scramble and shZeb1-1# and shZeb1-2# cells. All images were captured using a SIM microscope. The nucleus was counterstained with Hoechst dye. Scale bar = 3 μm. Boxed areas at a higher magnification showing detailed mitochondrial structures. **e** Quantifications of Frag, Tubu and Inter mitochondria in EML-scramble and shZeb1-1# and shZeb1-2#. **f** Transmission electron microscopic (TEM) images showing the mitochondrial morphology of EML-scramble, shZeb1-1# and shZeb1-2# cells. Scale bar = 0.2 μm. **g** Seahorse experiment showing the Oxygen consumption rate (OCR) of EML-scramble and shZeb1-1# and shZeb1-2# EML cells. **p < 0.001.

To verify whether Zeb1 can modulate mitochondrial morphology, we carefully observed mitochondrial structures in FACS-sorted Zeb1^−/low^ and Zeb1^+^ BM-LSKs using a structured illumination microscope (SIM) with an ultrahigh resolution. Intriguingly, SIM images (Fig. 6a) revealed an elongated and tubulated mitochondrial network in Zeb1^−/low^ LSKs. However, mitochondria in Zeb1^+^-LSKs were predominantly featured with spotted and fragmented morphology. We categorized mitochondrial morphologies into fragmented (Frag), tubulated (Tubu), and intermediate (Inter) mitochondria (Fig. 6b). These three types of mitochondria in Zeb1^−/low^ and Zeb1^+^-LSKs were quantified in Fig. 6c. We next sought to assess whether Zeb1 regulate mitochondrial morphology in EML cells. Compared with fragmented mitochondria in scramble cells, EML-shZeb1 cells showed elongated and tubulated mitochondria (Fig. 6d). The proportions of fragmented mitochondria dropped from 81% in scramble cells to 32–34% in EML-shZeb1 cells. In contrast, the percentages of tubulated mitochondria increased from 4.3% in scramble cells to 30.4–41.3% in shZeb1 cells (Fig. 6e). Transmission electron microscopy images further revealed an elongated and tubulated mitochondrial network in EML-shZeb1 cells compared to EML-scramble cells (Fig. 6f). We subsequently treated EML-shZeb1 cells with Benzyl isothiocyanate (BITC), a mitochondrial-fusion inhibitor, and found that stem-cell-associated-genes (Supplementary Fig. 6a-c) were significantly upregulated in response to BITC. These results suggested that Zeb1-mediated HSC stemness maintenance is intimately associated with a fragmented mitochondrial morphology.

We then asked whether downregulation of *Zeb1* can activate mitochondrial respiration in EML cells. The oxygen consumption rates (OCR) were determined by Seahorse experiments (Fig. 6g). The OCR was significantly increased when Zeb1 was downregulated, suggesting that downregulation of Zeb1 fuels OXPHOS activity. We also compared the mitochondrial morphology and quantified mitochondrial types in EML-vector and Zeb1-OE cells (Supplementary Fig. 6d-e). Overexpression of Zeb1 increased fragmented mitochondria from around 72–81%. These results demonstrated that Zeb1 endows EML cells with an immature fragmented mitochondrial morphology.

### Zeb1 suppresses mitochondrial fusion by directly targeting *Mfn2*

**Fig. 7:**
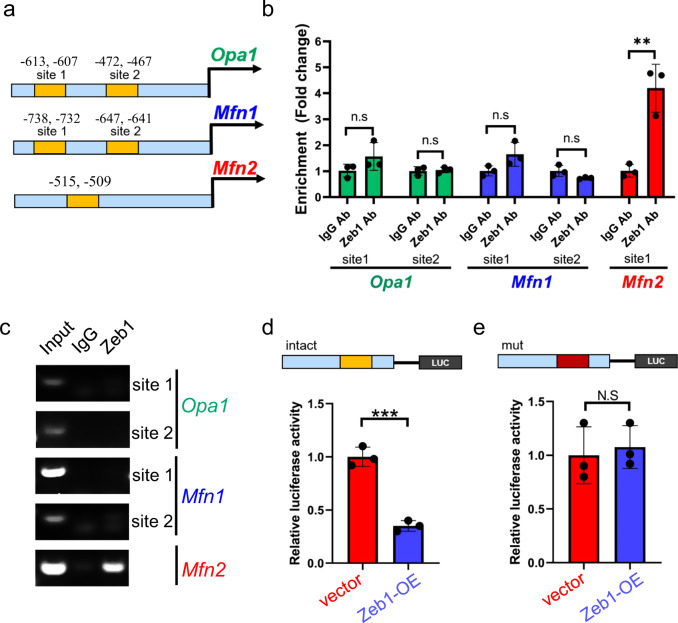
Zeb1 suppresses mitochondrial fusion by targeting *Mfn2*. **a** Identification of the putative bind sites of Zeb1 from start transcription site (STS) in the promoter regions of *Opa1* and *Mfn1* and *Mfn2*. **b** ChIP-qPCR reveals the binding of Zeb1 to the putative binding sites located at the *Opa1*, *Mfn1*, and *Mfn2* promote regions. **p < 0.001, N.S no significance. **c** Semi-quantitative ChIP showing the association of Zeb1 with the putative binding motifs at the *Opa1*, *Mfn1*, and *Mfn2* promote regions. **d** The intact *Mfn2* promoter region was cloned into a luciferase reporter construct and transfected into EML-vector and Zeb1-OE cells. A renilla plasmid was used as an internal control. Luciferase assays showing a significant transcriptional suppressive effect of Zeb1 on *Mfn2* transcription. ***p < 0.0001. **e** The *Mfn2* promoter with mutated binding site was cloned into a luciferase reporter construct and transfected into EML-vector and Zeb1-OE cells. Luciferase assays showing impaired suppressive effect of Zeb1 on *Mfn2* transcription.

Since overexpression of Zeb1 significantly decreased mitochondrial-fusion molecules (Fig. 5), we sought to determine whether Mfn1, Mfn2, and Opa1 have the putative binding motifs of Zeb1 in their promoter regions. To this end, the core promoter region of these genes from −1.5 kb upstream of the starting transcription site was blasted. Two putative Zeb1 binding sites were identified in the *Mfn1* and *Opa1* promoters, and one was found in the *Mfn2* promoter region (Fig. 7a). ChIP-qPCR assays only detected the binding of Zeb1 to the E-box motif in the *Mfn2* promoter region in EML cells (Fig. 7b, c). These results suggested that Zeb1 specifically occupies *Mfn2* promoter, but not *Mfn1* or *Opa1* promoters.

To assess the suppression of Zeb1 on *Mfn2* transcription, the *Mfn2* promoter was cloned, integrated into a luciferase construct, and transfected into EML-vector and Zeb1-OE cells. The significantly decreased luciferase activity was detected in the EML-Zeb1-OE cells compared to vector cells (Fig. 7d). We then assessed whether this binding site was required for the association of Zeb1 with the *Mfn2* promoter. To this end, we mutated this binding site in the *Mfn2* promoter luciferase construct. We found that the suppression of Zeb1 on *Mfn2* transcriptional activity was impaired when this binding motif was disrupted (Fig. 7e), suggesting that Zeb1 acts as a transcriptional suppressor of *Mfn2*.

Collectively, we demonstrated that Zeb1 suppresses mitochondrial fusion by suppressing *Mfn2* transcription, and thereby Zeb1 sustains a lower mitochondrial metabolism featuring reduced mitochondrial mass, decreased MMP, and fragmented mitochondrial morphology. These attributes together contribute to immature mitochondrial characteristics that benefit the long-term self-renewal and multilineage commitment of HSC.

## Discussion

We found that the EMT inducer Zeb1 plays an important role in bridging mitochondrial morphology and stemness maintenance in HSCs by suppressing Mitofusin-2. Using the *Zeb1-tdTomato*-reporter model and EML cells, we demonstrated that repression of *Mfn2* by Zeb1 contributes to fragmented mitochondria, thereby maintaining immature mitochondrial machinery with low mitochondrial mass, decreased MMP, reduced ROS levels, and dampened ETC and OXPHOS activities. Therefore, Zeb1 enables a low mitochondrial metabolic status that benefits HSC functions.

The EMT program has been employed by cancer cells to gain malignant phenotypes, including invasiveness and stemness in a wide spectrum of cancers [1, 2]. Our results demonstrated that HSC also relies on the EMT inducer Zeb1 for its maintenance. Previously, we found that Zeb1-expressing prostate basal epithelial cells are able to self-renew and process multipotency in producing all epithelial lineages, including luminal cells, basal cells, and neuroendocrine cells [6]. Now, we expand our scope to the hematopoietic system and reveal a broader function of Zeb1 in HSCs. Other EMT inducers, such as Slug have been reported to cooperate with Sox9 to sustain mammary stem-cell functions [24]. In HSCs, Twist1 was found to suppress voltage-gated calcium-channel CACNA1B mediated Ca^2+^ influx in the mitochondria [25]. Therefore, these studies, including ours, together provide a board implication that EMT program inducers may be employed by SSCs and CSCs to sustain stem-cell functions, although the mechanisms are likely to be differentially tailored in their details [26–28].

During the preparation of our manuscript, two elegant studies reported the role of *Zeb* transcription factors in the hematopoietic system [7, 8]. Almotiri et al. revealed that loss-of-*Zeb1* causes impaired cell-autonomous self-renewal and deficient multilineage differentiation. Particularly, they found that loss-of-*Zeb1* results in thymic atrophy due to impaired T cell lineage specification. The authors also found that *Zeb1*-KO HSCs display high levels of EpCAM, conferring these Zeb1^−/−^EpCAM^+^ HSCs with an enhanced survival advantage. Interestingly, these Zeb1^−/−^EpCAM^+^ HSCs also display altered metabolic pathways. Another study by Wang et al. paid particular attention to the synergistic role between *Zeb1* and *Zeb2* in sustaining hematopoietic lineage fidelity. In line with Almotiri’s study, they also found that loss-of-*Zeb1* results in cell-autonomous defects in multilineage differentiation, suggesting crucial roles of Zeb family transcription factors in governing stem-cell function and fine-tunes hematopoiesis [8].

Findings from these two elegant studies, together with our own work here, support a critical function of Zeb1 in HSC maintenance. Indeed, there exist discrepancies among these studies. These inconsistent results may be due to distinct experimental settings, for example, the animal models. The *Mx1*-Cre mouse model in those two studies not only introduces gene manipulations into the hematopoietic system but also in the stromal compartment. Similarly, *Tie2*-Cre-mediated knockout can introduce gene deletion in both hematopoietic stem/progenitor cells and endothelial cells. Administration of poly I: C may cause unexpected effects on HSC maintenance. Here, we employed a *Zeb1-tdTomato*-reporter line whose hematopoietic system is intact, and Zeb1 status is reflected by fluorescence at the physiological level, thereby excluding side effects that may hinder data interpretation.

The majority of results in our study were from adult BM-HSCs, but we also investigated the role of Zeb1 in FL-HSCs. Interestingly, our results suggest that Zeb1 is required in the maintenance of long-term stemness in both adult and fetal HSCs. Mechanistically, Zeb1 sustains BM-HSC functions via the Mitofusin-2/fragmented mitochondria axis. However, the exact mechanism by which Zeb1 regulates FL-HSC stemness is unclear. This raises an interesting question that how our major findings of Zeb1 in adult BM-HSCs also fit in the setting of FL-HSCs. A key feature of FL-HSCs is that they are highly proliferative cells compared to adult BM-HSCs [29]. As a versatile transcription factor, not merely limited to EMT, Zeb1 is recently reported to alleviate endoplasmic reticulum (ER) stress by reducing IRE1α signaling and Xbp1 splicing [30], suggesting that Zeb1 may protect replicative fetal HSCs from being impaired by biosynthesis-induced ER stress. Our results also demonstrated that Zeb1^+^-LSKs express higher superoxide dismutase 1/2 (*Sod1/2*) and catalase (*Cat*), indicating that Zeb1 may facilitate ROS clearance in fetal HSCs. Therefore, the function of Zeb1 in adult and fetal HSC maintenance may be achieved through distinct mechanisms in a context-dependent manner.

In summary, our study demonstrates that HSCs can employ Zeb1, a core EMT program inducer, to sustain stem-cell function by suppressing mitochondrial-fusion protein, Mitofusin-2, which bridges a previously unknown knowledge gap between an immature fragmented mitochondrial machinery and stemness maintenance.

## Materials and methods

### Mice

*Zeb1-tdTomato*-reporter mice with a C57BL/6 strain background were generated as previously reported [6]. The Global *Zeb1*-KO C57BL/6 mice were purchased from RIKEN-BRC (RBRC01923, Japan). The FL-HSCs collected from embryonic fetal livers at E13.5 were used for experiments. All animal experiments were performed according to the instruction of Animal Research Ethics Committee of Ren Ji Hospital.

### Cell culture

EML cells were commercially available from American Type Culture Collection with Short Tandem Repeat validation. EML cells were cultured with Iscove’s modified Dulbecco’s medium (Gibco) with 10% fetal bovine serum (Gibco), supplemented with 20% conditional medium of BHK-21 cells, which were transfected with stem-cell factor expressing lentivirus, 100 U/ml penicillin (Gibco), and 100 μg/ml streptomycin sulfate (Gibco). Other procedures are presented in Supplementary Methods and Materials

## Supplementary information

Supplemental Figure 1

Supplemental Figure 2

Supplemental Figure 3

Supplemental Figure 4

Supplemental Figure 5

Supplemental Figure 6

Supplemental methods and materials

Supplemental Figure legend

Supplemental Table 1: Antibodies, reagents and kits

Supplemental Table 2: primers

RNA-seq gene expression matrix

uncropped WB data

check-list

## Data Availability

The RNA-seq dataset is available in the National Omics Data Encyclopedia with databank accession number OEP003535. All experimental results generated in this study are displayed in this published article and its Supplementary Information files.
