# Supplementary figures and images for: Zeb1 sustains hematopoietic stem cell functions by suppressing mitofusin-2-mediated mitochondrial fusion
Source: Cell Death Dis. 2022 Aug 25;13(8):735. doi: 10.1038/s41419-022-05194-w (PMC9411618; doi:10.1038/s41419-022-05194-w)

# Supplemental Figure 1

a WT *Zeb1* allele

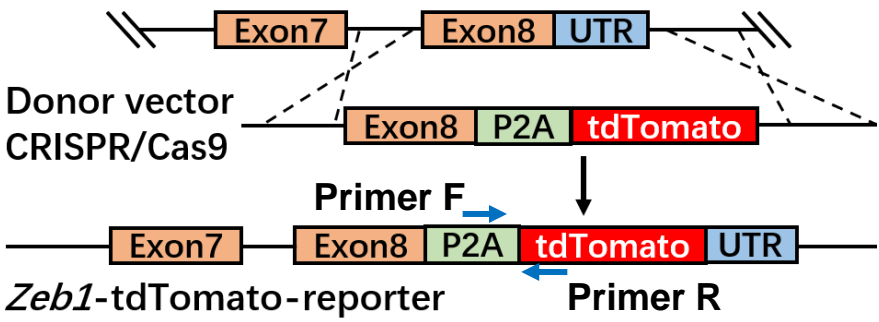

b

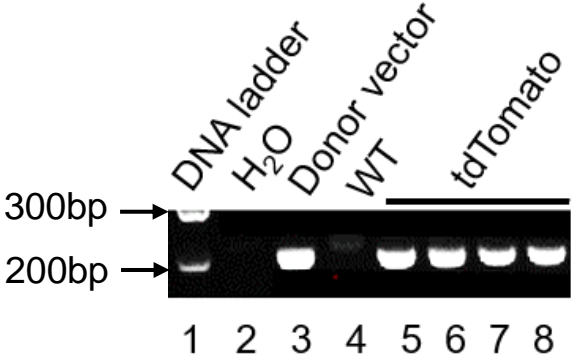

c

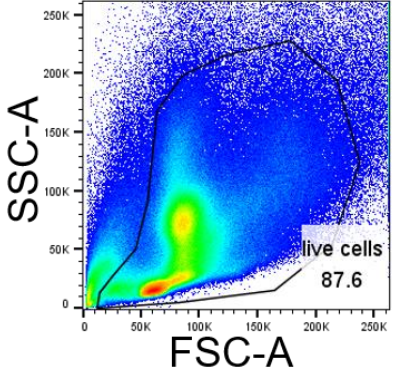

d

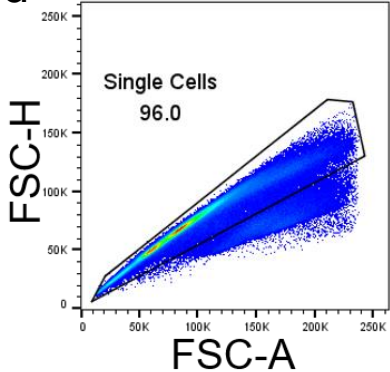

e

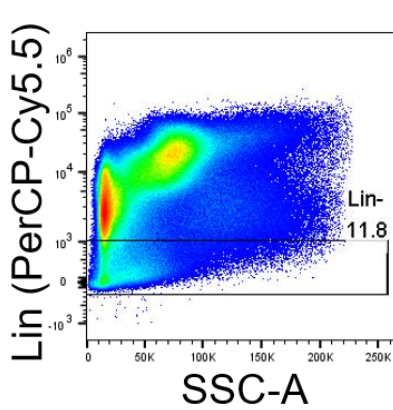

f

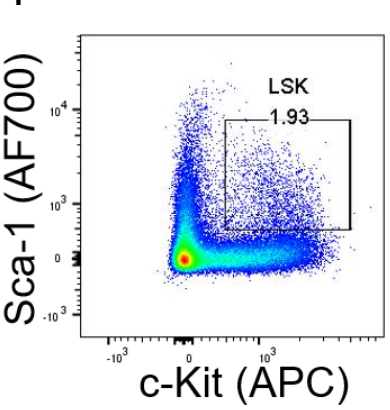

Supplement: Supplementary file 1 — Supplemental Figure 1 [file 41419_2022_5194_MOESM1_ESM.pdf]

# Supplemental Figure 3

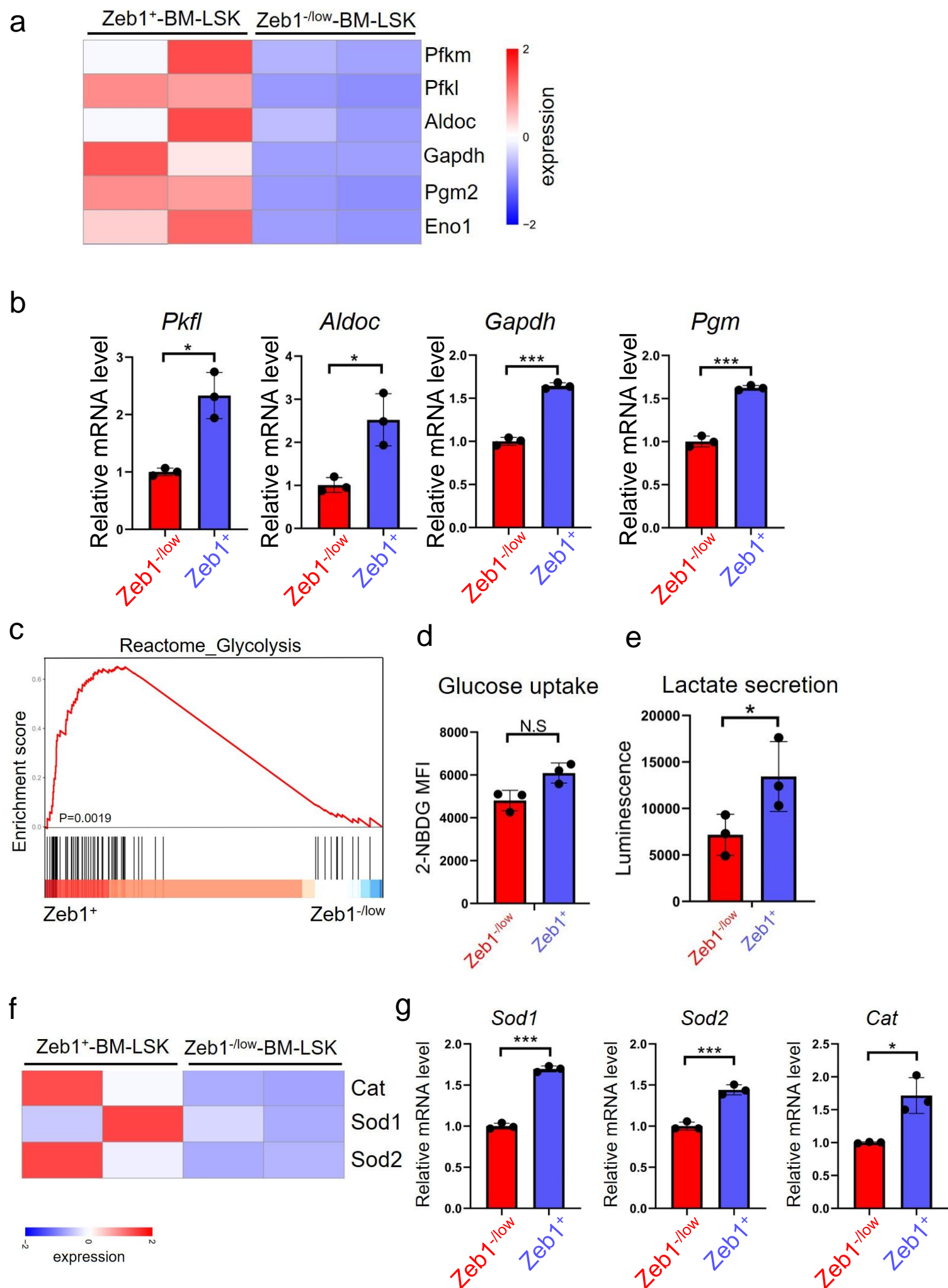

Supplement: Supplementary file 3 — Supplemental Figure 3 [file 41419_2022_5194_MOESM3_ESM.pdf]

Supplemental Figure 4

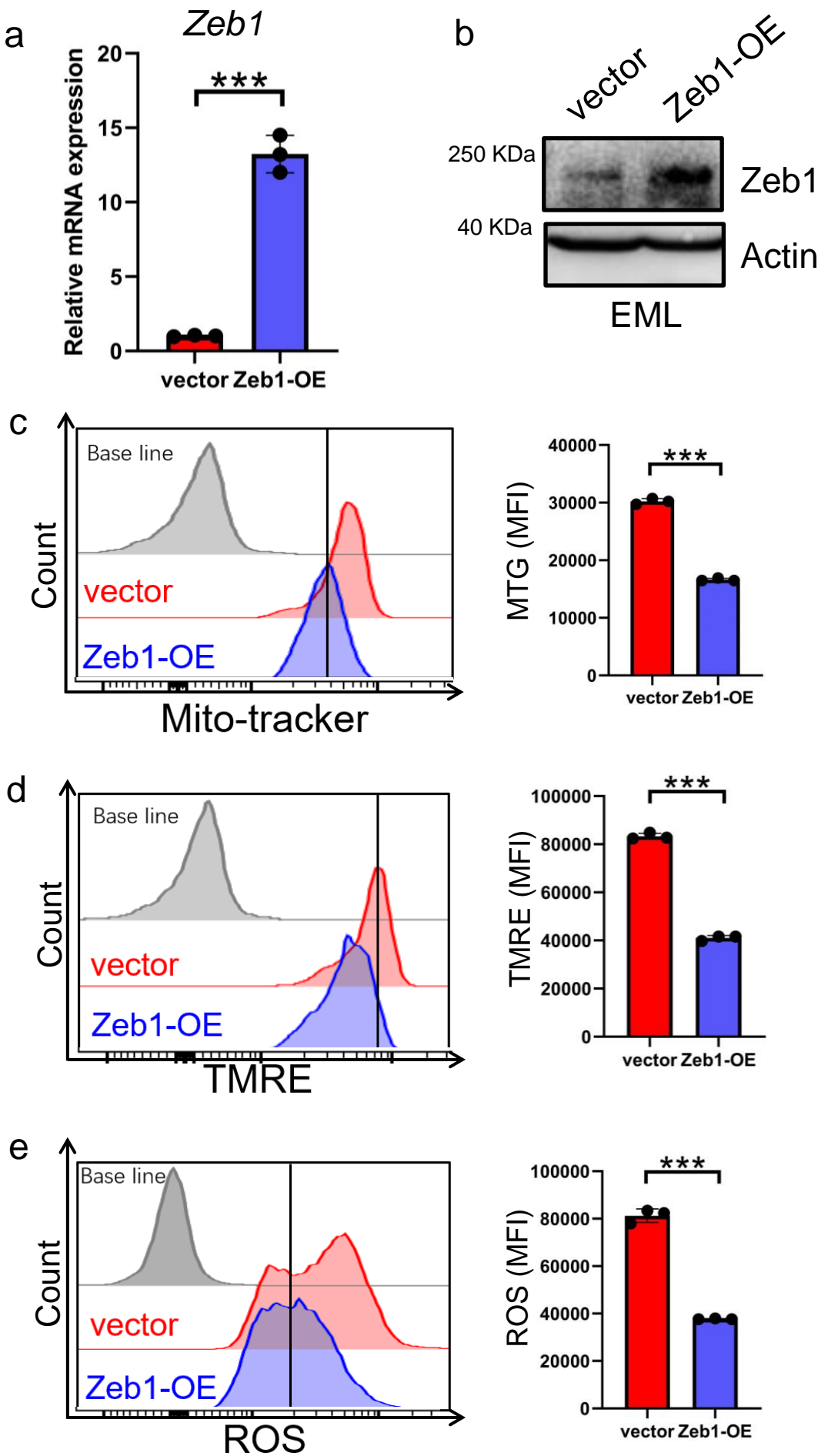

Supplement: Supplementary file 4 — Supplemental Figure 4 [file 41419_2022_5194_MOESM4_ESM.pdf]

Supplemental Figure 5

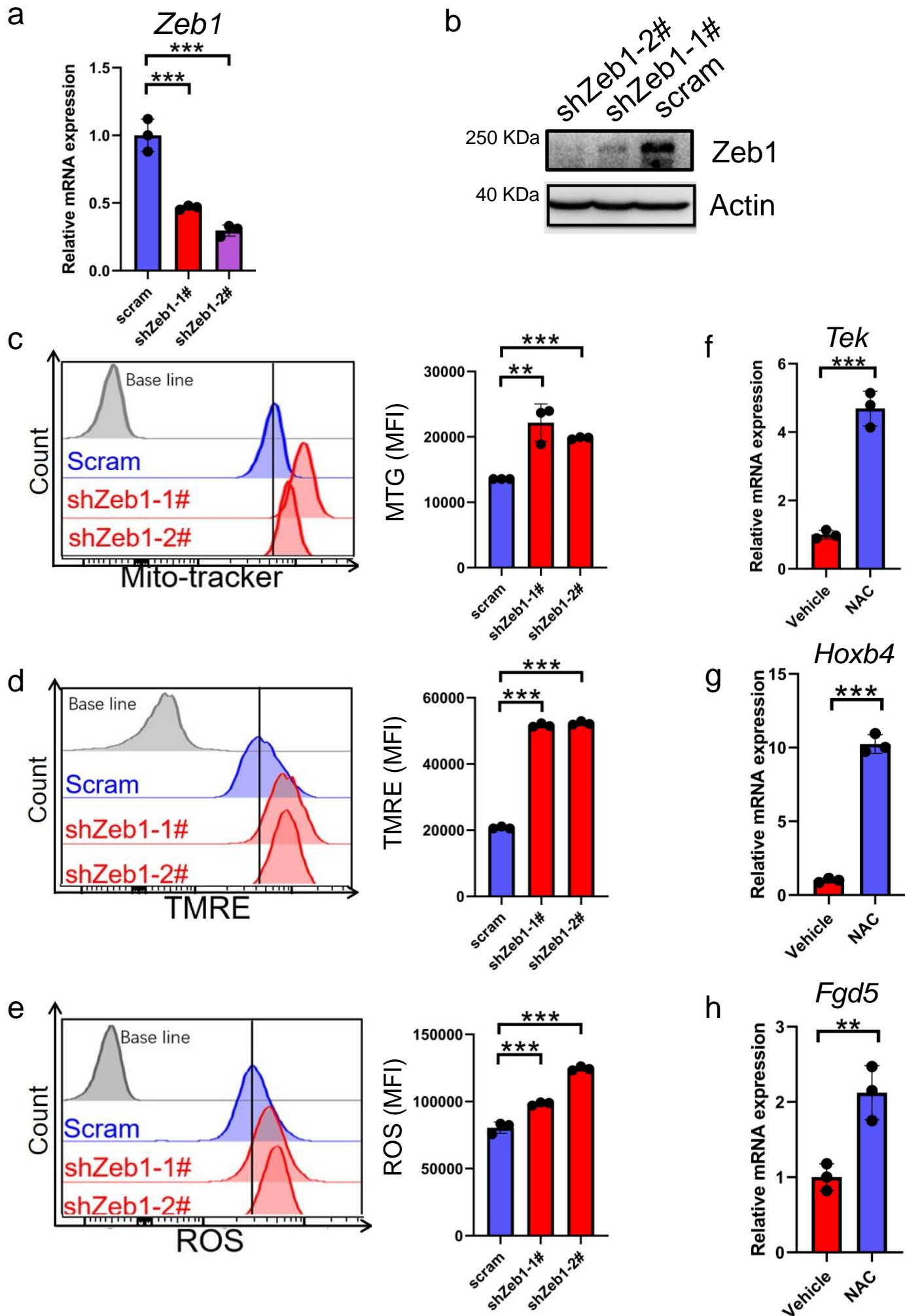

Supplement: Supplementary file 5 — Supplemental Figure 5 [file 41419_2022_5194_MOESM5_ESM.pdf]

## Supplemental Figure 6

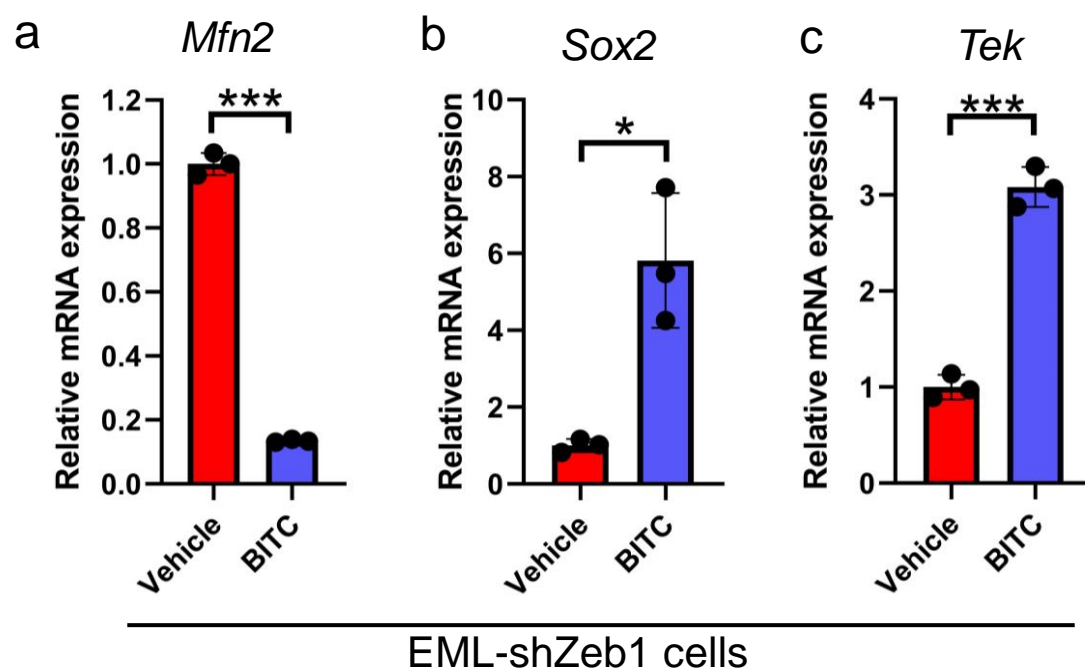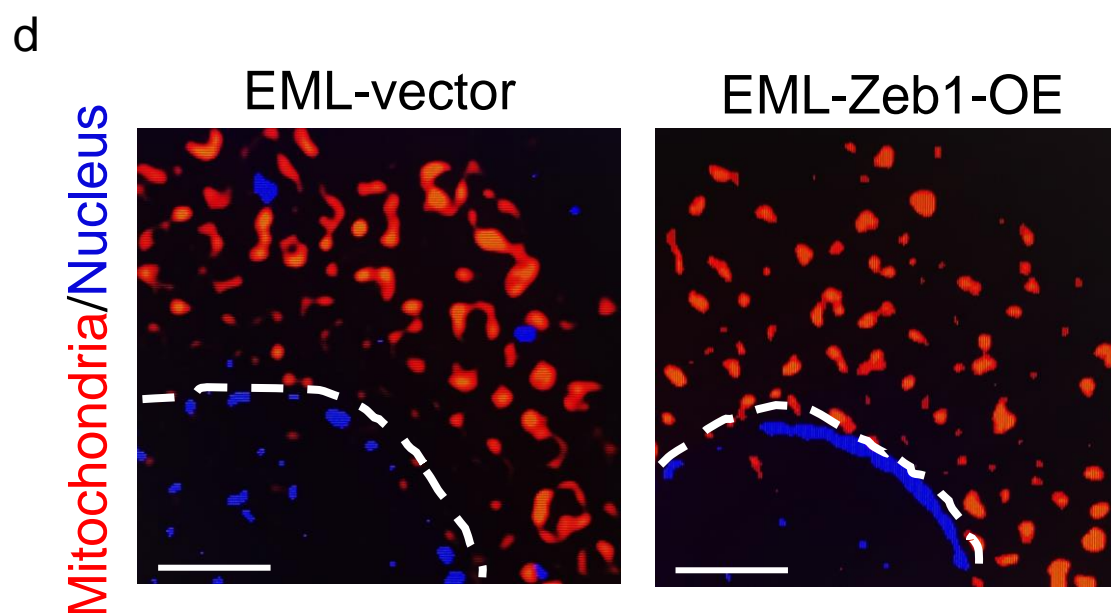

**e**

|         | ○ Frag | ● Inter | ● Tubu |
|---------|--------|---------|--------|
| Vector  | 72%    | 21.3%   | 6.7%   |
| Zeb1-OE | 81%    | 14.9%   | 4.1%   |

EML cells

Supplement: Supplementary file 6 — Supplemental Figure 6 [file 41419_2022_5194_MOESM6_ESM.pdf]

Supplemental uncropped WB data

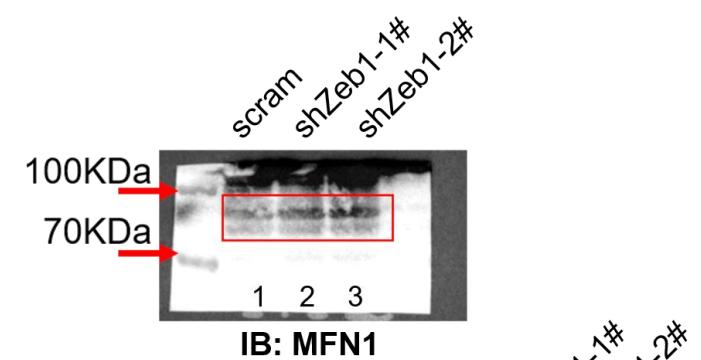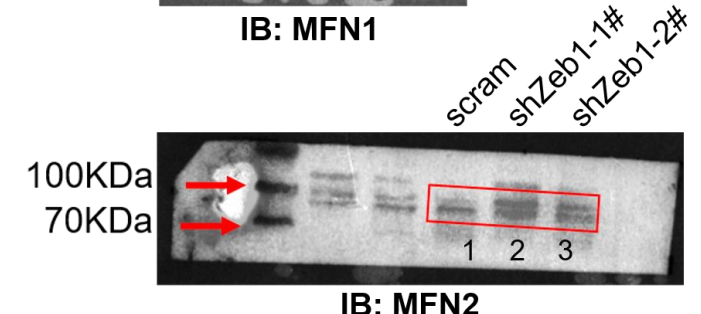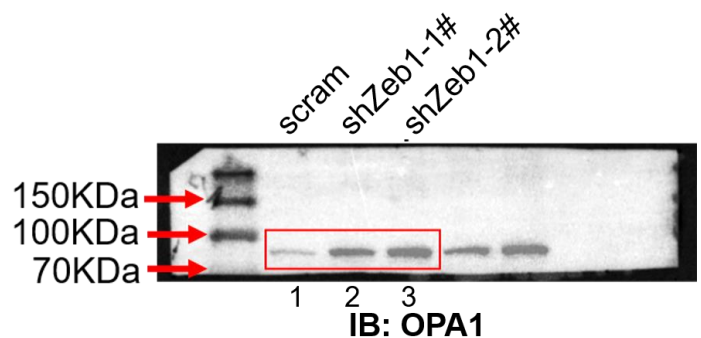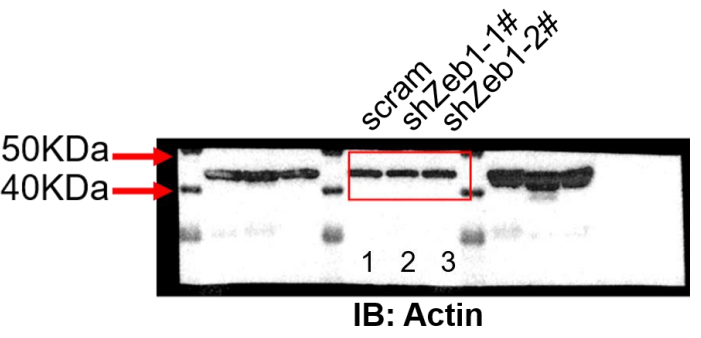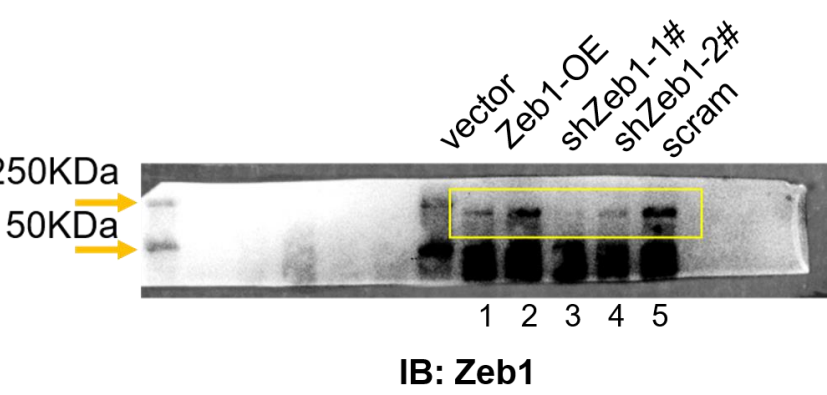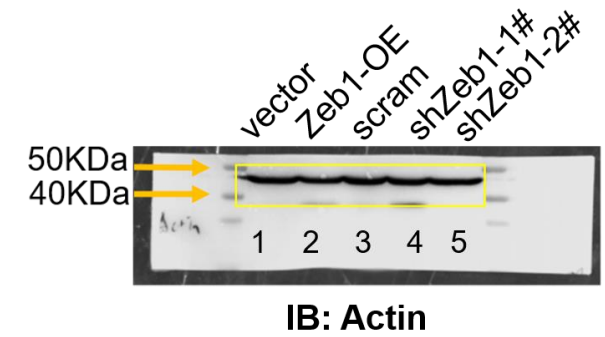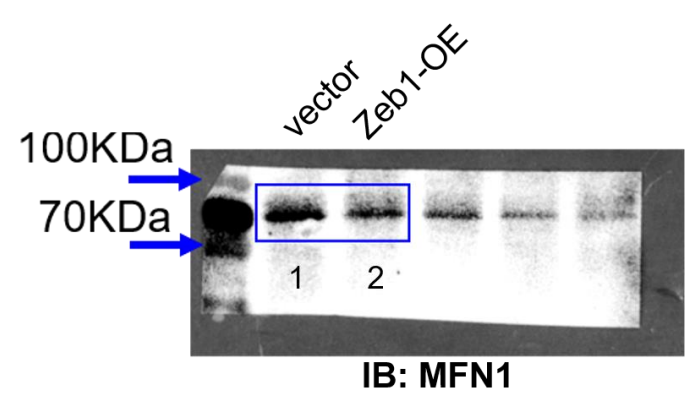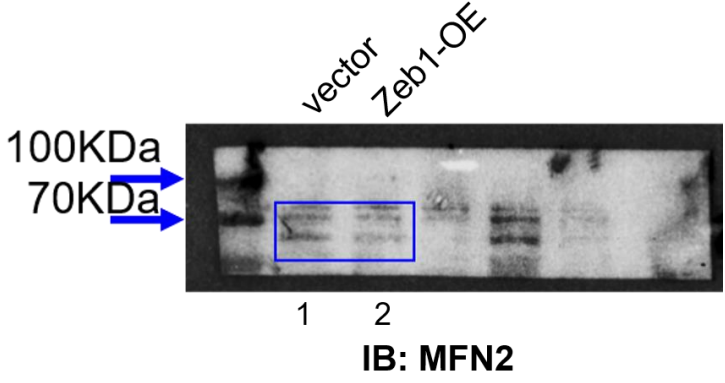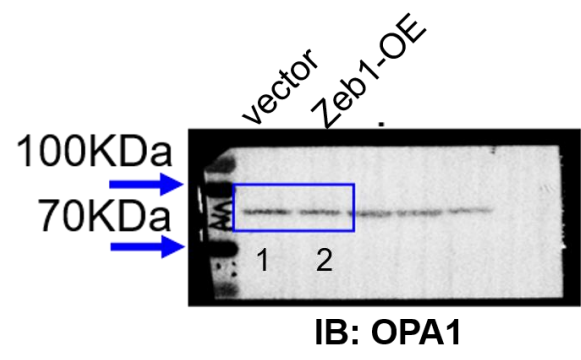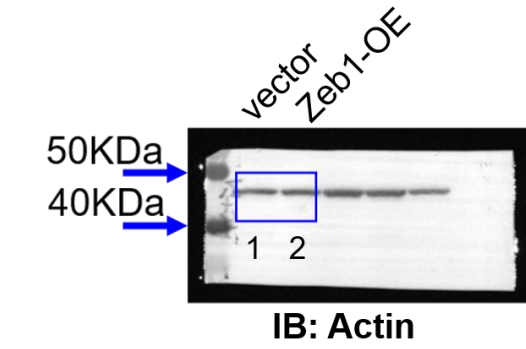

Supplement: Supplementary file 12 — uncropped WB data [file 41419_2022_5194_MOESM12_ESM.pdf]
