# Supplementary material for: Zeb1 sustains hematopoietic stem cell functions by suppressing mitofusin-2-mediated mitochondrial fusion
Source: Cell Death Dis. 2022 Aug 25;13(8):735. doi: 10.1038/s41419-022-05194-w (PMC9411618; doi:10.1038/s41419-022-05194-w)

Supplemental Figure 2

a

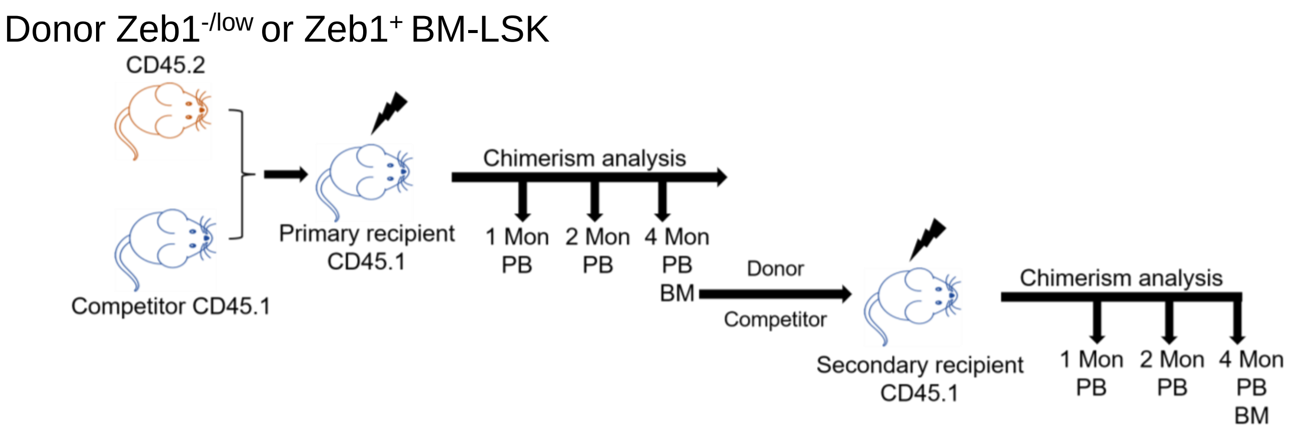

b

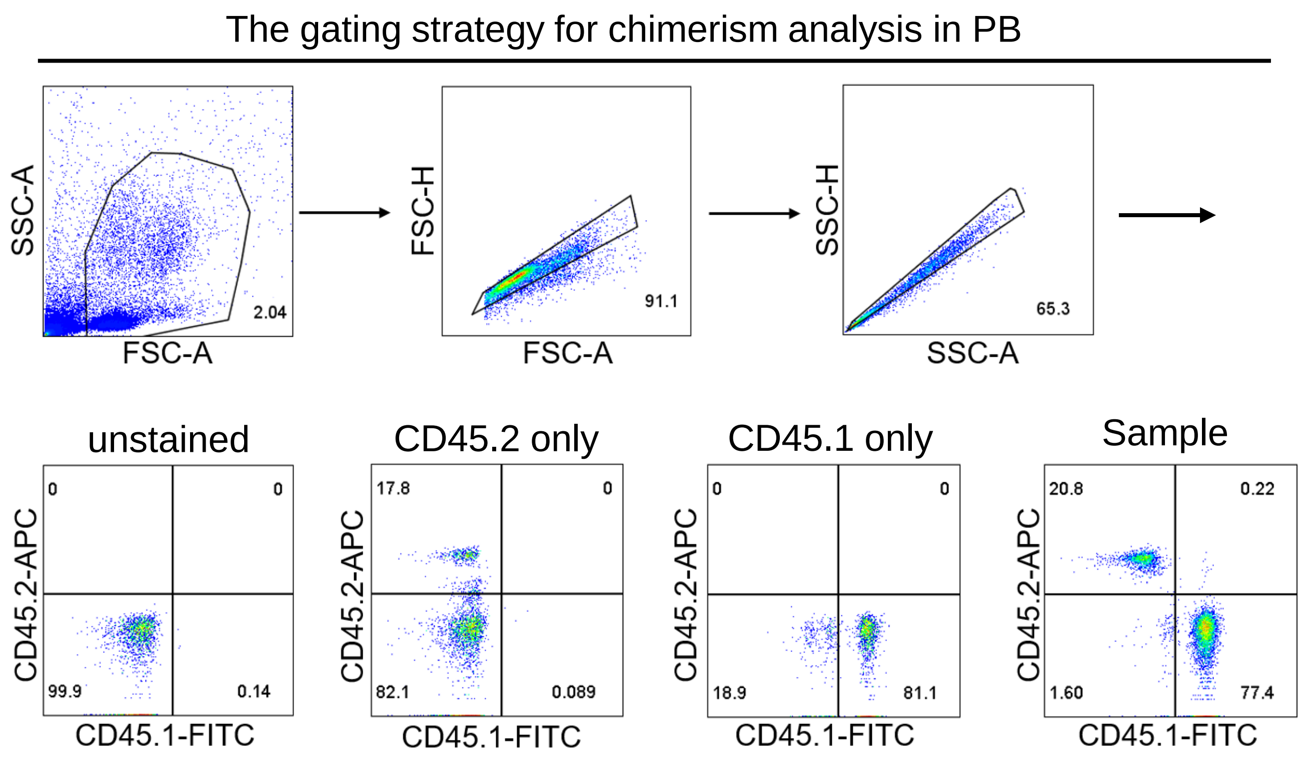

c

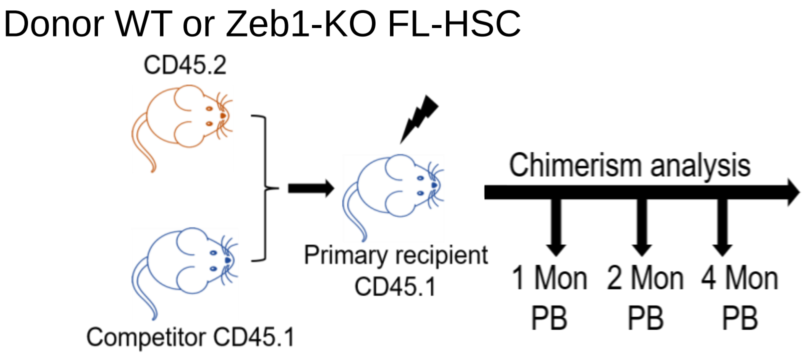

Supplement: Supplementary file 2 — Supplemental Figure 2 [file 41419_2022_5194_MOESM2_ESM.pdf]
