# Supplementary material for: Zeb1 sustains hematopoietic stem cell functions by suppressing mitofusin-2-mediated mitochondrial fusion
Source: Cell Death Dis. 2022 Aug 25;13(8):735. doi: 10.1038/s41419-022-05194-w (PMC9411618; doi:10.1038/s41419-022-05194-w)
Supplement: Supplementary file 7 — Supplemental methods and materials [file 41419_2022_5194_MOESM7_ESM.docx]

**Supplemental Methods and Materials**

**Definition of Zeb1^-/low^ and Zeb1^+^-LSK cells in BM**

*Zeb1-tdTomato* reporter mice (6-8 weeks old, male) with C57BL/6 background were euthanized and femurs and tibias were collected and carefully crushed by pestle and mortar in the buffer of PBS supplemented with 2% FBS (FACS buffer). The bone marrow (BM) cell suspensions were then filtered through the 70 μm BD falcon cell strainers. The BM cells were subsequently transferred to 50 mL EP tubes with 20 mL pre-cold FACS buffer and centrifuged at 300 g for 5 min. Then, the BM cells were resuspended with 500 μL FACS buffer and stained with flow antibodies based on manufacturer’s guidelines. To purify LSK cells, we used a lineage antibody cocktail, together with c-Kit-APC, and Sca1-Alexa Fluor (AF) 700 flow antibodies. In detail, the lineage antibody cocktail includes biotinylated B220 (B cells), CD3e (T cells), Ter119 (erythrocytes), Mac1 (monocytes) and Gr-1 (granulocytes) flow antibodies. A PerCP-Cy5.5 streptavidin was also used to label the biotinylated lineage antibody cocktail with a fluorochrome. By gating the PerCP-Cy5.5 negative cells, we focused on the lineage negative (Lin-) BM progenitor and hematopoietic stem cell population. Sca-1-AF700 and c-Kit-APC double positive cells, which were gated from the Lin- population, were designated as LSK cells. In this study, LSK cells that fall in the 20% maximal tdTomato fluorescence value were defined as Zeb1^+^ LSKs, and those in the 20% minimal tdTomato fluorescence were defined as Zeb1^-/low^ LSKs. Co-stained with CD150-FITC and CD48-APC-Cy7, CD150^+^CD48^-^LSK cells were designated as HSCs. BD FACS Aria Ⅱ instrument was used for cell sorting, and BD LSR Fortessa X-20 instrument was utilized for flow cytometric analysis. Data were analyzed using Flowjo software.”

**Peripheral blood (PB) analysis**

PB were collected and transferred to EDTA-precoated Eppendorf tubes on a roller to avoid coagulation. PB were incubated with red blood cell lysis buffer at room temperature (RT) for 10 min, then washed with phosphate buffer saline (PBS, pH=7.4) before staining immunophenotypic markers. Flow antibodies were incubated at 4℃ for 30 min in the dark room. Cells were washed with FACS buffer (PBS with 2% Fetal Bovine Serum, FBS) and filtered with cell strainer (BD Falon, 70 μm) prior to flow cytometry analysis.

**Bone marrow (BM) analysis**

Mice were euthanized and then femurs and tibias were collected and carefully crushed by pestle and mortar. BM cells were transferred to 50 mL EP tubes with 20 mL pre-cold FACS buffer, filtered, washed and centrifuged at 300 g for 5 min. Lineage (B220, CD3e, Ter119, Gr-1 and Mac1) negative BM progenitor cells were either enriched by magnetic separation (MACS, Miltenyi, biotech) or FACS sorting based on well-established cell surface markers. Lin^-^Sca-1^+^c-Kit^+^ BM cells represent LSK cells, and CD150^+^CD48^-^LSK were used to define HSCs. As to the analysis of committed lineages, the red blood cells were lysed, washed and stained for prospective lineage markers before FACS analysis.

**Isolation of fetal liver HSCs**

For global Zeb1-KO mice, we isolated their fetal liver HSCs via magnetic-activated cell sorting (MACS)-based separation assay. Briefly, we isolated E13.5 embryos and placed them in pre-cold PBS in a sterile 6-cm dish. The fetal livers were carefully extracted using sharp tweezers. Subsequently, the fetal livers were homogenized and filtered through a 70 μm cell strainer and resuspended in FACS buffer to generate single cell suspensions. Biotinylated lineage antibody cocktail (including B220, CD3e, Ter119, Gr-1, Mac1, CD31 and CD326) was added to single cell suspension at a dilution of 1: 100. Mix them well by pipetting gently and incubate at RT for 1 hour. Centrifuge at 200×g for 10 min and discard supernatant. Resuspend cells with FACS buffer and add 20 μL biotin-magnetic beads into single cell suspension with 10^7^ cells. Mix them well by pipetting gently and incubate at RT for 30 min. After magnetically labeled, cells were passed through a MACS-column which is placed in a MACS-separator. Magnetically labeled cells were retained within the column, and unlabeled cells passed through the column. These unlabeled cells were HSCs and collected for experiments.

**BM transplantation assays**

Recipient mice (CD45.1) were lethally irradiated (9.5 Gy). For primary transplantation, 200 Zeb1^-/low^ and Zeb1^+^ LSKs isolated from *Zeb1-tdTomato*-reporter mice (CD45.2) were mixed with 2×10^5^ BM supporting cells (CD45.1) respectively and transplanted into recipient mice via intravenous tail vein (i.v.) injection. PB was analyzed for donor chimerism at indicated time points. BM was analyzed for the lineage commitment and prepared for the secondary transplantation. To further determine the capability of Zeb1^-/low^ and Zeb1^+^ LSKs of lineage reconstitution in the secondary recipients, 200 Zeb1^-/low^ and Zeb1^+^ LSKs (CD45.2) isolated from the primary recipients were mixed with 2×10^5^ total BM cells (CD45.1) and transplanted through i.v. injection. The following procedures of donor chimerism and lineage reconstitution were performed in the similar way as described. The authors also performed transplantation experiments using Zeb1-KO and WT fetal liver HSCs. For fetal liver HSC transplantations, 10^4^ donor cells and 10^4^ competitor cells were mixed and transplanted into recipient mice.

**Hematopoietic stem cells colony forming unit (HSC-CFU) assay**

For *in vitro* CFU assays, 250 Zeb1^-/low^ and Zeb1^+^ LSKs were purified via FACS sorting and seeded on methylcellulose based semi-solid medium (HSC007, R&D) in response to cytokine stimulation. The colonies were enumerated and characterized based on their unique morphology. For *in vivo* CFU-Spleen (CFU-S) assays, 10^5^ single cell suspensions of Zeb1^-/low^ and Zeb1^+^ LSKs were i.v. transplanted into lethally irradiated recipients. Spleens were collected from recipient mice and fixed in 4% paraformaldehyde at 14 days after transplantation. The colonies were counted and representative images were captured. The authors also conducted CFU-S experiments using Zeb1-KO and WT fetal liver HSCs.

**Metabolic analyses**

**Mitochondrial mass, membrane potential, intracellular ROS level, and 2-NBDG glucose uptake**

Cells were washed and incubated with either of these mitochondrial dyes including Mito-Tracker^TM^ Green (30nM, M7514, Invitrogen), Mito-Tracker^TM^ Red (30nM, M7512, Invitrogen), TMRE (100nM, T669, Invitrogen) and carboxyl-H2DCFDA (20 μM, C400, Invitrogen) for 30 min at 37℃. Detailed procedures were based on manufacturers’ instructions. Cells were washed and continued with either low cytometry analysis or counterstained with Hoechst (C1022, Beyotime) for microscopic inspection. Mitochondrial mass was determined by Mitochondrial Tracker Green or Red dyes. Mitochondrial membrane potential was assessed by TMRE. Intercellular ROS was measured by carboxyl-H2DCFDA. Glucose uptake rate was assessed by a 2-NBDG based glucose uptake assay kit (Cayman chemical, Cat: 600470).

**Immunofluorescence (IF)**

Staining for mitochondrial dyes were conducted based on manufacturer’s instructions. For IF staining of Tomm20, the cells were incubated with the primary antibodies overnight at 4℃. Cells were washed with PBS and then incubated with secondary antibodies for 1 hour. Cells were subsequently washed and counterstained with either Hoechst or DAPI (Molecular probes). Images of mitochondrial structures were captured using Structured Illumination Microscope (SIM, Nikon).

**Plasmids and viruses**

The cDNA sequence of Zeb1 was cloned into a lentiviral vector LeGO (Plasmid # 27358, Addgene) fused with a green fluorescent protein (GFP). Two short hairpin RNA vectors shZeb1-1# and shZeb1-2# were generated by cloning 5’- TGCCAGCAGACCAGACAGTAT-3’ and 5’- TAGCTCACATATAAGCAGTAA-3’ into a lentiviral vector GV112 (Shanghai Genechem, China) respectively. The WT *Mfn2* promoter from -1 to -1500bp was cloned into a lentiviral vector (Lenti-V2-Blast) with a luciferase cassette. A putative Zeb1 binding site was identified within this region. In the mutated luciferase promoter construct, the putative Zeb1 binding site CACCTG was replaced with GAAGTG. EML cells were infected these lentiviruses for 48 hours and then 3 μg/mL puromycin was added into the culture medium for 2 weeks to generate stable expressing cell lines including EML-vector, EML-Zeb1-OE, EML-scramble, EML-shZeb1-1# and 2#.

**Immunoblotting assay**

Whole cell lysates of EML-vector, EML-Zeb1-OE, EML-scramble, EML-shZeb1-1# and 2# were prepared using RIPA lysis buffer (10mM Tris-HCl, pH=8.0, 1mM EDTA, 0.5mM EGTA, 1% Triton X-100, 0.1% Sodium Deoxycholate, 0.1% SDS, 140mM NaCl). The extracted proteins were separated by electrophoresis and incubated with primary antibodies as indicated followed by three washes and incubated with horseradish peroxidase conjugated secondary antibodies. The images were assessed using chemiluminescence detector (Bio-Rad).

**Transmission Electron Microscopy (TEM)**

Briefly, cells were fixed in 2.5% glutaraldehyde, 2.5% paraformaldehyde in 0.1 M solution sodium cacodylate buffer (pH=7.4). Sectioning and imaging were conducted according to the Life Science Microscopy Center at Shanghai Jiao Tong University.

**RNA sequencing (RNA-seq)**

RNA from FACS sorted Zeb1^-/low^ and Zeb1^+^ LSKs were extracted using RNA-easy Micro RNA extraction kit (Qiagen). RNA quality and quantity were determined by Agilent 2100 Bioanalyzer RNA 6000 kit (Agilent Technologies). 100 pg RNA were used to produce cDNA with Smart-seq II protocol. The cDNA library was then purified, washed and quantified using Qubit High Sensitivity DNA kit (Thermo Fisher). Next, 50 pg cDNA were tagged followed by amplification. The cDNA library was sequenced using a 2×75 bp paired end dual index read format on a Illumina HiSeq-4000 based on manufacturer’s guidelines (Illumina Inc). Sequencing was performed by running 150 cycles. The differentially expressed genes and transcripts were filtered using R package Ballgown. Heatmap and Gene Ontology analysis were performed with DEG in R or python environment for statistical analysis and graphic images.

Similarly, RNA-seq was also performed on EML-scramble and EML-shZeb1 cells. The raw data is accessible @https://www.biosino.org/node/ and the access number is OEP003535.

**Bioinformatics analysis**

For RNA-seq, sequencing FASTQ files were uploaded and processed with RNA seq Alignment Application (Illumina Inc) to obtain raw data of reads counts for detected genes. Heatmap graphic of gene expression were generated using R package pHeat map. Gene Set Enrichment Analysis (GSEA) was performed using GSEA 4.0 software with 1000 gene set permutations using gene ranking matrix t-test with customized symbols.

**Quantitative PCR (qPCR) assay**

Total RNA from cultured EML cells or FACS sorted LSK cells were extracted using TRIZOL (invitrogen) according to manufacturer’s instructions. Then, RNAs were reverse-transcribed into cDNA using random Oligo dT primers and reverse transcriptase buffer (Takara). Real time quantitative PCR experiments were performed using SYBR Green Master Mix (Takara) on a Step One Plus Real-time PCR system (Applied Biosystems).

**Luciferase reporter assay**

*Mfn2* promoter luciferase reporters with intact and mutated putative Zeb1 binding site were used to transfect EML cells together with a Renilla expressing construct. Luciferase activities were measured by a dual luciferase detection system (Promega) at 72 hours post transfection.

**ChIP-qPCR assay**

2×10^7^ EML-Zeb1-OE cells were harvested and fixed with 1% PFA. ChIP experiments were performed using the Simple ChIP ™Enzymatic Chromatin IP Kit (#9003, CST) according to the manufacturer’s instruction. The antibody against Zeb1 was purchased from Novus Biological (NBP1-05987).

**Oxygen consumption assay**

EML-scramble, EML-shZeb1-1# and shZeb1-2# cells were seeded onto Cell Tak-coated 24-well seahorse plate with the density of 2×10^5^ cells per well in a total of 100 μL stem cell media supplemented with pyruvate sodium (0.5 mM, Gibco). The plate was then spun at 200 g for 1 min and immediately incubated at 37℃ for 30 min. 500 μL stem cell media was carefully added to each well and then incubated at 37℃ for 30 min. Next, samples were loaded into the Seahorse XF24 Extracellular Flux Analyzer (Seahorse Biosciences). The resting respiration of cells were determined in the first place. Cells were then treated with oligomycin (1 μg/μL) to assess the non-phosphorylating oxygen consumption rate (OCR). Subsequently, cells were treated with FCCP (2 μM) to measure the maximal OCR. Finally, antimycin A (1 μM) were added to detect the extramitochondrial OCR. Each step was taken a 2-min-interval followed by a 2-min-mixing and 2-min-incubation.

**Lactate secretion assay**

The lactate secretion assay was performed using the Lactate-Glo-assay kit (Promega, J5021) based on the manufacturers’ instructions.

**Statistical analysis**

Results are presented as the means ± SD. for N independent experiments or N mice, as shown in the figure legends. Statistical analyses were performed using Student’s t test using GraphPad Prism 8 software. P value of <0.01 was considered to have statistical significance (*P < 0.01, **P < 0.001, ***P < 0.0001).
