# Supplementary material for: Zeb1 sustains hematopoietic stem cell functions by suppressing mitofusin-2-mediated mitochondrial fusion
Source: Cell Death Dis. 2022 Aug 25;13(8):735. doi: 10.1038/s41419-022-05194-w (PMC9411618; doi:10.1038/s41419-022-05194-w)
Supplement: Supplementary file 8 — Supplemental Figure legend [file 41419_2022_5194_MOESM8_ESM.docx]

**Supplemental Figure legend**

**Supplemental Figure 1**

**(a)** The schematic strategy of the generation of *Zeb1-tdTomato* reporter mice using CRISPR/Cas9 technology. UTR: untranslated region; P2A: porcine teschovirus-1 2A. A pair of primer was designed for genotyping as indicated by blue arrows.

**(b)** Genotyping results of *Zeb1-tdTomato* reporter mice and their WT littermates. Distilled H_2_O was used as a negative control. Donor vector served as a positive control.

**(c-f)** The flow cytometric analysis showing the LSK gating strategy in a step-by-step manner. Briefly, the viable cells were gated **(c)**, followed by singlet cells inclusion **(d)** and then the exclusion of lineage positive cells **(e)**. Finally, the Lin^-^Sca1^+^c-Kit^+^ (LSK) subsets were gated as LSKs **(f)**.

**Supplemental Figure 2**

**(a)** The schematic strategy of the two round competitive BM transplantation and reconstitution experiments using Zeb1^+^ and Zeb1^-/low^ LSKs from *Zeb1-tdTomato* reporter mice.

**(b)** The flow cytometric results showing the gating strategy for chimerism analysis in PB in a step-wise manner. Briefly, viable cells were gated and followed by single cell inclusion. An unstained sample was shown as a negative control. Single staining of either CD45.2-APC (CD45.2 only) or CD45.1-FITC (CD45.1 only) and a representative sample were shown accordingly.

**(c)** The schematic strategy of competitive BM transplantation and reconstitution experiment using WT and Zeb1-KO fetal liver HSCs.

**Supplemental Figure 3**

1. The heat map showing key glycolytic enzyme genes were enriched in BM Zeb1^+^-LSKs as compared to Zeb1^-/low^ counterparts.
2. RT-qPCR results confirming glycolytic enzyme genes including *Pfkl*, *Aldoc*, *Gapdh*, and *Pgm2* were upregulated in Zeb1^+^-LSKs.
3. The GESA plot indicates the enrichment of glycolysis related hallmarks in Zeb1^+^-LSKs.
4. 2-NBDG based glucose uptake assay showing Zeb1^+^-LSK cells have higher capacity of glucose uptake that Zeb1^-/low^ counterparts.
5. Lactate-Glo-assay showing Zeb1^+^-LSK cells can produce higher amount of lactate than Zeb1^-/low^ LSK cells.
6. The heat map showing ROS scavenging related genes were enriched in BM Zeb1^+^-LSKs as compared to Zeb1^-/low^ counterparts.
7. RT-qPCR results confirming ROS scavenging related genes including *Sod1*, *Sod2* and *Cat* were upregulated in Zeb1^+^-LSKs.

**Supplemental Figure 4**

**(a-b)** RT-qPCR **(a)** and immunoblotting data **(b)** confirming the ectopic expression of Zeb1 in EML cells.

**(c-e)** Representative FACS plot and MFI of Mito-tracker **(c)**, TMRE **(d)** and ROS **(e)** of EML-vector and EML-Zeb1-OE cells.

**Supplemental Figure 5**

**(a-b)** RT-qPCR **(a)** and immunoblotting data **(b)** confirming the knockdown efficiency of Zeb1 by short hairpin shZeb-1# and shZeb1-2# in EML cells.

**(c-e)** Representative FACS plot and MFI of Mito-tracker-green (MTG) **(c)**, TMRE **(d)** and ROS **(e)** of scramble and shZeb1-1# and shZeb1-2# EML cells.

**(f-h)** RT-qPCR results showing stem cell associated genes including *Hoxb4*, *Fgd5* and *Tek* were increased in response to the treatment of the antioxidant, N-acetyl-L-cysteine (NAC, 0.5μM, MCE) in EML-shZeb1-1# cells.

**Supplemental Figure 6**

**(a-c)** RT-qPCR results showing two typical stem cell associated genes including *Tek* and *Sox2* were increased in response to the treatment of a mitochondrial fusion inhibitor, Benzyl isothiocyanate (BITC, 0.5μM, MCE) in EML-shZeb1-1# cells. The significantly decreased *Mfn2* level confirming that mitochondrial fusion was indeed blocked by BITC.

**(d)** Representative images of Mito-Tracker-Red in EML-vector and EML-Zeb1-OE cells by IF staining. The images were captured using Structured illumination microscopy (SIM). Nucleus was counterstained with Hoechst dye. Scale bar = 1 μm.

**(e)** The percentage of tubulated (black), fragmented (white) and intermediated (gray) mitochondria in EML-vector and EML-Zeb1-OE cells.
