# Supplementary material for: Zeb1 sustains hematopoietic stem cell functions by suppressing mitofusin-2-mediated mitochondrial fusion
Source: Cell Death Dis. 2022 Aug 25;13(8):735. doi: 10.1038/s41419-022-05194-w (PMC9411618; doi:10.1038/s41419-022-05194-w)
Supplement: Supplementary file 9 — Supplemental Table 1: Antibodies, reagents and kits [file 41419_2022_5194_MOESM9_ESM.docx]

**Supplemental Table 1**

**1.1 Antibodies used for FACS analysis and sorting**

| **Antibodies** | **Lot No.** | **Vendor** |
| --- | --- | --- |
| Mouse Hematopoietic Lineage Biotin Panel | 88-7774-75 | eBioscience |
| PerCP-Cy5.5 Streptavidin | 45-4317-82 | eBioscience |
| Sca-1-APC | 17-5981-82 | eBiosceince |
| c-Kit-FITC | 11-1171-85 | eBiosceince |
| c-Kit-PE-Cy7 | 25-1171-82 | eBioscience |
| CD150-Alexa-Fluor647 | 115910 | Biolegend |
| CD48-APC-Cy7 | 561242 | BD |
| CD45.2-APC | 17-0454-82 | eBioscience |
| CD45.1-FITC | 11-0453-85 | eBioscience |
| Gr-1-biotin | 13-5931-75 | eBioscience |
| CD3e-biotin | 13-0031-85 | eBioscience |
| B220-biotin | 13-0452-75 | eBioscience |

**1.2 Antibodies used for immunoblotting (IB), immunofluorescence (IF) and ChIP**

| **Antibodies** | **Lot No.** | **Vendor** |
| --- | --- | --- |
| Zeb1 (IB) | D80D3 | Cell signaling technology |
| Zeb1(ChIP) | NBP105987 | Novus biotechnology |
| Mfn1(IB) | A9880 | Abclonal |
| Mfn2(IB) | 9482S | Cell signaling technology |
| Opa1(IB) | A9833 | Abclonal |
| Actin (IB) | AC026 | Abclonal |
| Tomm20 (IF) | sc-17764 | Santa Cruz |

**1.3 Fluorescent dyes and kits**

| **Dyes and reagents** | **Lot No.** | **Vendor** |
| --- | --- | --- |
| Mito Tracker Green (MTG) | M7514 | Invitrogen |
| Mito Tracker Red | M7512 | Invitrogen |
| TMRE | T669 | Invitrogen |
| carboxyl-H2DCFDA | C400 | Invitrogen |
| Oligomycin | HY-N6782 | MCE |
| FCCP | HY-100410 | MCE |
| Antimycin A | D5030 | Sigma |
| Benzyl isothiocyanate (BITC) | HY-77813 | MCE |
| N-acetyl-cysteine (NAC) | HY-B0215 | MCE |
| Glucose-uptake cell- based assay kit | 600470 | Cayman chemical |
| Lactate Glo assay kit | Promega | J5021 |
