# Supplementary material for: Zeb1 sustains hematopoietic stem cell functions by suppressing mitofusin-2-mediated mitochondrial fusion
Source: Cell Death Dis. 2022 Aug 25;13(8):735. doi: 10.1038/s41419-022-05194-w (PMC9411618; doi:10.1038/s41419-022-05194-w)
Supplement: Supplementary file 10 — Supplemental Table 2: primers [file 41419_2022_5194_MOESM10_ESM.docx]

**Supplemental Table 2**

**1 Primer sequence for Zeb1-tdTomato reporter mice genotyping**

Forward

5’-TCCCCATCCCCTCTAAACCT-3’

Reverse

5’-CCCTGTTGCTTTGGTAGTGA-3’

**2 Primer sequence for conventional Zeb1-KO mice genotyping**

Forward-1

5’-AGCACTATTCTCCGCTACTCCAC-3’

Forward-2

5’-AACCGTGCATCTGCCAGTTTGAG-3’

Reverse

5’-ACCGCACCTGGTTTACGACACTC-3’

**3 Primer sequences for RT-qPCR (5’-3’)**

| *Zeb1* | Forward: CCACTGTGGAGGACCAGAAT |
| --- | --- |
|  | Reverse: CTCGTGAGGCCTCTTACCTG |
| *Actin* | Forward: GGCTGTATTCCCCTCCATCG |
|  | Reverse: CCAGTTGGTAACAATGCCATGT |
| *tdTomato* | Forward: ACCAAGCTGGACATCACCTC |
|  | Reverse: GCGCATGAACTCTTTGATGA |
| *Bmi1* | Forward: ATCCCCACTTAATGTGTGTCCT |
|  | Reverse: CTTGCTGGTCTCCAAGTAACG |
| *Sox2* | Forward: GCGGAGTGGAAACTTTTGTCC |
|  | Reverse: CGGGAAGCGTGTACTTATCCTT |
| *Hoxb4* | Forward: CAGAGCGATTACCTACCCAGC |
|  | Reverse: ATTGGGGTTTACCGTGCTCAC |
| *Tal1* | Forward: ACTAGGCAGTGGGTTCTTTGG |
|  | Reverse: CCCGGCTGTTGGTGAAGAT |
| *Fgd5* | Forward: CGATCCAGAGACGGATGGGA |
|  | Reverse: GTGCCCTGTTATCACTGTTGT |
| *Tek* | Forward: TCTGATGCCGAAACATCCCTC |
|  | Reverse: CCTCCAGTGGATCTTGGTGC |
| *Stk1* | Forward: CTGGACTCCGAGACCTTATGC |
|  | Reverse: CAAGCTGGATCACATTCCGAT |
| *Mfn1* | Forward: CCTACTGCTCCTTCTAACCCA |
|  | Reverse: AGGGACGCCAATCCTGTGA |
| *Mfn2* | Forward: TGACCTGAATTGTGACAAGCTG |
|  | Reverse: AGACTGACTGCCGTATCTGGT |
| *Opa1* | Forward: CGACTTTGCCGAGGATAGCTT |
|  | Reverse: CGTTGTGAACACACTGCTCTTG |
| *Dnm1l* | Forward: TTACGGTTCCCTAAACTTCACG |
|  | Reverse: GTCACGGGCAACCTTTTACGA |
| *Fis1* | Forward: AGGCTCTAAAGTATGTGCGAGG |
|  | Reverse: GGCCTTATCAATCAGGCGTTC |
| *Mff* | Forward: TACACCGAAGGTATTAGTCAGCG |
|  | Reverse: ACTTGCATTATCACACTGGCAT |
| *Ppargc1a* | Forward: TATGGAGTGACATAGAGTGTGCT |
|  | Reverse: GTCGCTACACCACTTCAATCC |
| *Atg3* | Forward: ACACGGTGAAGGGAAAGGC |
|  | Reverse: TGGTGGACTAAGTGATCTCCAG |
| *Map1l3b* | Forward: TTATAGAGCGATACAAGGGGGAG |
|  | Reverse: CGCCGTCTGATTATCTTGATGAG |
| *Sod1* | Forward: AACCAGTTGTGTTGTCAGGAC |
|  | Reverse: CCACCATGTTTCTTAGAGTGAGG |
| *Sod2* | Forward: CAGACCTGCCTTACGACTATGG |
|  | Reverse: CTCGGTGGCGTTGAGATTGTT |
| *Cat* | Forward: GGAGGCGGGAACCCAATAG |
|  | Reverse: GTGTGCCATCTCGTCAGTGAA |

**4 Primer sequences for ChIP-qPCR (5’-3’)**

| *Mfn1* promoter  site 1# | Forward: GCCAATAGGAGATCCGAAGC |
| --- | --- |
|  | Reverse: GACACTCACTCCGTCCCT |
| *Mfn1* promoter  site 2# | Forward: TAGGAATCCACCCGGCTGG |
|  | Reverse: CTGGTGTGCGTGAAAAGAAG |
| *Opa1* promoter  site 1# | Forward: GAGACTTTGACAAGGGCCA |
|  | Reverse: GCTGTAGGTTTGGGATAAACAC |
| *Opa1* promoter  site 2# | Forward: CCTACAGCTTAAATGTCAGAAG |
|  | Reverse: GAATGTTTATTAGGAGCAGCCA |
| *Mfn2* promoter  site | Forward: TCCTGGGACTGAGTGAAAATT |
|  | Reverse: GGTGAAGTAATTGTCCTTCAGT |
